# Cell Membrane Biophysics as a Therapeutic Interface for Nanomedicine: From Disease-Associated Remodeling to Translational Qualification

**DOI:** 10.3390/cells15171525

**Published:** 2026-08-24

**Authors:** Yueming Yin, Dan Fan, Ling An, Yi Liu, Yaling Liu

**Affiliations:** 1 School of Engineering, Dali University, Dali 671003, China; yuemingyin@stu.dali.edu.cn (Y.Y.); fandan@stu.dali.edu.cn (D.F.); anling@stu.scu.edu.cn (L.A.); 2 Precision Medicine Translational Research Center, West China Hospital, Sichuan University, Chengdu 610041, China; 3 Department of Bioengineering, Lehigh University, Bethlehem, PA 18015, USA

**Keywords:** cell membrane biophysics, therapeutic interface, membrane-guided nanomedicine, disease-associated remodeling, biomimetic nanocarriers

## Abstract

**Highlights:**

**What are the main findings?**

- Disease-associated changes in lipid organization and fluidity, membrane mechanics, receptor organization, glycocalyx architecture, and bioelectric states create context-dependent therapeutic opportunities and delivery barriers.
- An interface-centered framework connects disease-remodeled membrane states to mechanistically matched nanomedicine strategies and a function-centered pathway for translational qualification.

**What are the implications of the main findings?**

- Candidate membrane cues should be prioritized when they are accessible, disease-enriched, and functionally linked to recognition, transport, or therapeutic response.
- Clinical translation requires measurable membrane-dependent function supported by interaction evidence, membrane-specific quality and potency, context-matched validation, in vivo pharmacology and safety, and reproducible manufacturing.

**Abstract:**

Nanomedicine has yielded clinically useful platforms, including liposomes, albumin-bound nanoparticles, and lipid nanoparticles; yet, many systems translate poorly because of nonspecific biodistribution, limited target-site accumulation, inefficient cellular uptake and intracellular delivery, immune clearance, and off-target toxicity. These bottlenecks are often shaped at cell membrane interfaces, where therapeutic materials are recognized, retained, internalized, or cleared and may elicit unsafe responses. Here, we frame cell membrane biophysics as a therapeutic interface for nanomedicine. We examine how lipid organization and fluidity, mechanics, electrochemical state, glycocalyx architecture, and membrane protein identity shape recognition, adhesion, endocytosis, fusion, trafficking, immune responses, and drug release. We assess how disease-associated membrane remodeling can create candidate therapeutic entry points and delivery barriers across cancer, neurodegeneration, inflammation, infection, and vascular disease. We then analyze receptor-mediated targeting, lipid-domain-associated uptake, membrane-coated nanocarriers, engineered extracellular vesicles, and hybrid platforms, with explicit context-of-use definitions and design boundaries. Finally, we propose translational qualification through function-linked critical quality attributes, mechanism-relevant potency assays, context-matched models, in vivo pharmacology and immune safety, scalable manufacturing, and regulatory evaluation. Progress will depend less on descriptive membrane mimicry than on measurable, reproducible, and qualified membrane-dependent functions.

## 1. Introduction

Nanomedicine has expanded therapeutic options through clinically translated nanocarriers, including liposomes, albumin-bound nanoparticles, and lipid nanoparticles (LNPs), which can stabilize payloads and alter pharmacokinetics and tissue exposure in selected contexts [1,2]. Extracellular vesicles (EVs) and biomimetic vesicles extend this landscape by leveraging native membrane composition and intercellular communication but face challenges in scale-up, cargo loading, heterogeneity, and biodistribution [3]. Translation remains limited by sequential barriers that restrict target-site accumulation, cellular uptake, and intracellular delivery while promoting nonspecific biodistribution, immune clearance, and off-target toxicity [1,4]. Many of these constraints are shaped at the cell membrane, a dynamic lipid–protein–carbohydrate interface where nanocarrier recognition, adhesion, uptake, and membrane perturbation affect transport, safety, and therapeutic performance [5,6]. The membrane is therefore not merely a passive boundary but a therapeutic interface that can couple—or uncouple—nanoscale design from selective cellular access, intracellular action, and biological safety.

Classical membrane biology underpins this interface view. The fluid mosaic model established the plasma membrane as a dynamic, selectively permeable lipid–protein assembly [7], later refined by evidence for glycoconjugate-rich surface layers, lipid domains, and nanoscale heterogeneity [8,9,10]. Membrane composition, lipid–protein interactions, and higher-order organization preserve integrity and permeability while regulating receptor signaling, trafficking, and transport [11,12,13,14]. At cellular and model-membrane levels, lipid-domain organization, membrane mechanics, and nanoparticle–membrane interaction energetics influence binding, wrapping, and membrane perturbation [15,16,17], with consequences for endocytic entry, immune recognition, and cytotoxicity [18,19,20]. Living membranes are therefore multiscale, biologically active interfaces.

At this interface, target-membrane features—including lipid organization, receptor nanoclustering, mechanical state, and glycocalyx-associated glycosylation [10,21,22]—and carrier properties—including stiffness, surface chemistry, ligand accessibility, and corona formation [23,24,25]—jointly shape receptor engagement, protein adsorption, cellular uptake, and in vivo fate. Recent perspectives on immune-compatible aggregation-induced-emission nanoplatforms further highlight biomimetic cloaking, protein adsorption and corona effects, and interface engineering as important design considerations at the nano–bio interface [26]. Cell membrane-coated systems may combine source-derived recognition and reduced-clearance functions with engineered targeting, but their performance depends on preserving membrane integrity and function during manufacturing [27,28]. EV-based drugs similarly require control of identity, heterogeneity, cargo loading, potency, and nonclinical behavior before translational claims are justified [29]. Biophysical and imaging methods can complement endpoint uptake and viability assays only when their readouts are linked to the claimed membrane-dependent function.

We use three terms to distinguish mechanistically distinct levels. Disease-associated membrane remodeling denotes changes intrinsic to or directly coupled to target-cell membranes, including lipid organization, membrane–cortex mechanics, electrochemical state, membrane protein organization, and glycocalyx architecture. Nanocarrier–cell membrane interactions denote binding, insertion, wrapping, endocytosis, or fusion determined jointly by membrane state and carrier properties. Membrane-derived carrier functions denote activities retained or engineered in cell membrane coatings and EVs, including source-dependent recognition, immune interaction, and cargo transfer. By contrast, extracellular matrix (ECM) mechanics, hemodynamics, and protein corona formation are treated as tissue-, flow-, or nano–bio-context modifiers; blood–brain barrier (BBB) transport is treated as a barrier-level process that includes but is not reducible to endothelial membrane transport.

Membrane biophysics also links disease mechanisms to treatment responsiveness. Across disease contexts, membrane remodeling can involve altered mechanics and ion channel activity and can influence virus–cell membrane fusion and immune recognition, although dominant phenotypes vary by disease and cell type [30,31,32,33]. These features may create candidate therapeutic vulnerabilities and context-dependent delivery constraints, not universally validated delivery determinants. Their relevance depends on disease enrichment, receptor or glycocalyx accessibility, functional coupling to binding, cellular entry, or trafficking, and compatibility with carrier mechanics and surface chemistry [10,23,24,34]. The same complexity also imposes translational boundaries: disease heterogeneity, nonspecific biodistribution, immune clearance, off-target exposure, manufacturing scalability, and batch variability can uncouple promising membrane-level mechanisms from reproducible clinical benefit.

Previous reviews have examined nanoparticle–membrane and nano–bio interactions, drug-carrier biointerface engineering, cell membrane-coated nanocarriers, EV delivery, and broader translational challenges [1,3,5,27,29,34]. Focused analyses have also addressed cancer cell membrane-coated platforms and homotypic tumor recognition, including fabrication, immune-related functions, and major limitations [35]. Yet, these perspectives remain largely interaction-, carrier-, platform-, or translation-centered and rarely integrate the disease-remodeled target membrane with a unified evidence hierarchy. The remaining need is to connect disease-associated target-membrane remodeling to mechanistically defensible therapeutic opportunities, distinguish them from associative phenotypes and delivery barriers, match them to appropriate interventions, and define how the resulting membrane-dependent functions should be experimentally and translationally qualified.

**Figure 1:**
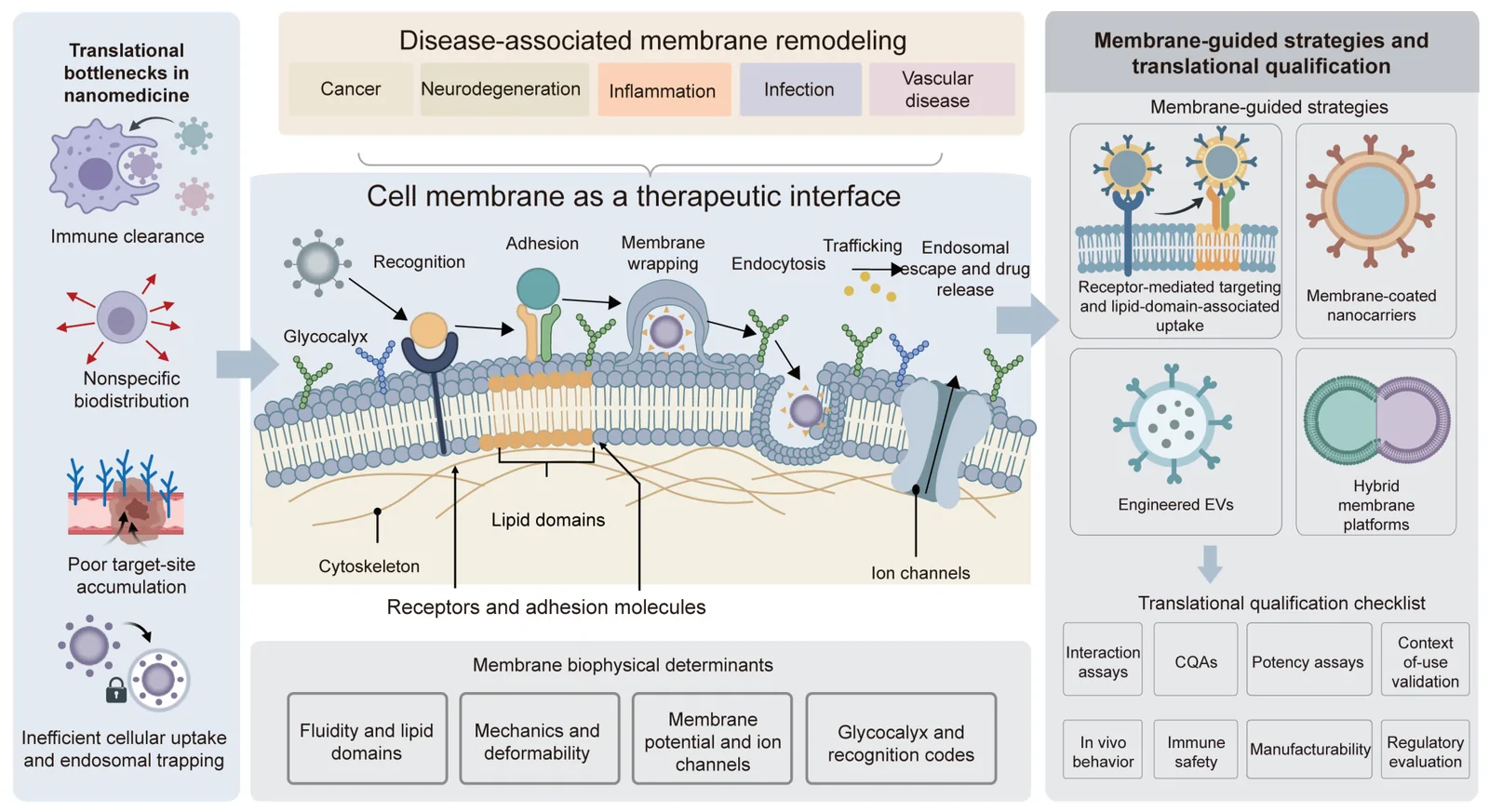
Cell membrane biophysics as a therapeutic interface for membrane-guided nanomedicine. The schematic connects major translational bottlenecks with membrane-associated recognition, adhesion, membrane wrapping, endocytosis, trafficking, endosomal escape, and drug release. These events are influenced by membrane fluidity and lipid-domain organization, mechanics and deformability, membrane potential and ion channels, and glycocalyx architecture and recognition codes. Disease-associated remodeling across cancer, neurodegeneration, inflammation, infection, and vascular disease can create candidate therapeutic entry points and delivery barriers. Membrane-guided strategies include receptor-mediated targeting, lipid-domain-associated uptake, membrane-coated nanocarriers, engineered extracellular vesicles, and hybrid membrane platforms. Their qualification requires interaction assays, critical quality attributes, potency assays, context-of-use validation, in vivo behavior, immune safety, manufacturability, and regulatory evaluation. CQAs, critical quality attributes; EVs, extracellular vesicles.

We therefore organize cell membrane biophysics within a mechanism–remodeling–strategy–qualification framework rather than another carrier classification. Evidence level, disease and context-of-use relevance, strategy compatibility, and design boundaries determine whether a membrane feature is actionable. Figure 1 summarizes this framework. Section 2 and Section 3 define membrane architecture and the biophysical determinants of therapeutic interactions; Section 4 and Section 5 evaluate disease-remodeled entry points and barriers and match them to membrane-guided strategies; and Section 6 links claimed functions to interaction evidence, membrane-specific critical quality attributes, mechanism-relevant potency assays, context-matched models, in vivo pharmacology and immune safety, and manufacturing and regulatory control.

## 2. From a Passive Barrier to a Therapeutic Interface: Conceptual Evolution of Cell Membrane Biophysics

The cell membrane has evolved conceptually from a passive, selectively permeable boundary to a dynamic, heterogeneous, and therapeutically relevant interface. Classical descriptions emphasized cellular integrity, molecular exchange, pH, and osmotic balance, whereas the fluid mosaic model established a dynamic lipid–protein bilayer rather than a static boundary [7]. Subsequent work revealed spatial organization, nanoscale heterogeneity, and lipid–protein interactions that regulate recognition, signaling, and transport [8,9,11], extending rather than replacing the selective-barrier concept. Because these properties also determine how therapeutic materials contact, enter, and perturb cells, membrane organization provides the basis for an active interface that can be read, remodeled, and engineered.

### 2.1. From Selective Permeability to Dynamic Membrane Architecture

Selective permeability remains fundamental: by regulating the exchange of ions, nutrients, metabolites, and waste, the membrane preserves electrochemical gradients, cellular pH, and osmotic homeostasis, whereas uncontrolled permeability disrupts metabolism, signaling, and compartmentalized reactions. The fluid mosaic model reframed this boundary as a dynamic lipid–protein architecture that maintains selective permeability while permitting lateral molecular mobility [7]. Lipids form the bilayer matrix, whereas membrane proteins and lipid–protein interactions provide functional specificity by coupling permeability to signaling and transport [11,14].

### 2.2. From Molecular Composition to Spatially Organized Membrane Domains

The fluid mosaic model established a dynamic bilayer, but subsequent work showed that biological membranes are spatially organized, laterally heterogeneous, and actively regulated [8]. Lipid asymmetry, phase behavior, cholesterol-dependent order, and lipid–protein interactions jointly shape membrane structure, domain organization, and function [12,14]. In living mammalian plasma membranes, the cytoplasmic leaflet is substantially more unsaturated, whereas the exoplasmic leaflet is more tightly packed and less diffusive; related asymmetry also occurs in transmembrane protein domain organization [36]. The leaflets nevertheless communicate through composition- and property-dependent coupling: in asymmetric model membranes, outer-leaflet lipid composition can strongly influence lipid diffusion in the opposing leaflet, whereas structural order may remain weakly coupled [37]. Leaflet asymmetry and transbilayer coupling therefore add a vertical dimension to membrane organization.

Membrane proteins further diversify this architecture by mediating signaling, transport, and other specialized functions, and many clinically exploited drug targets are membrane proteins [11,38]. Lipids and membrane-associated proteins form rafts, nanodomains, and other functional domains that regulate molecular mobility and signaling [15]. Membrane–cortex coupling and cortical actin constrain this organization, while integrins connect pericellular matrix cues to membrane organization and signaling [22,39]. Glycolipids and glycoproteins add spatial and biochemical specificity through glycocalyx-dependent organization and recognition [10]. Membrane function therefore emerges from the lateral and transbilayer organization of lipids, proteins, and carbohydrates rather than from composition alone.

### 2.3. From Dynamic Biophysics to Therapeutic Interface Engineering

Membrane fluidity, lipid nanoenvironments, and domains shape receptor engagement and membrane protein activity [12,40]; membrane tension and related mechanical properties influence deformation, wrapping, and carrier–cell contact [16,17]; ion channel regulation contributes to electrochemical signaling and responsiveness [41]; and membrane proteins and glycocalyx-associated glycans regulate accessibility, immune recognition, and material–cell compatibility [10,19]. Together, these properties determine whether nanomaterials merely contact the cell surface or establish functionally relevant interactions. Similar bulk uptake values can nevertheless represent different surface-bound, membrane-inserted, or internalized nanoparticle populations [42].

Nanomedicine performance therefore cannot be inferred from carrier composition, payload design, or target expression alone [34]. Nanomaterials engage membranes through electrostatic, hydrophobic, and ligand–receptor-mediated interactions [5,6] and may subsequently undergo endocytic uptake and intracellular trafficking [42,43]. Poorly controlled interactions can instead perturb plasma or endosomal membranes, trigger inflammatory responses, or cause cytotoxicity [20,44,45]. Membrane-level design becomes therapeutically meaningful only when interface engagement is linked to productive uptake, release, immune compatibility, or another intended function.

## 3. Biophysical Determinants of Membrane–Therapeutic Interactions

Cell membrane biophysics governs how therapeutic systems encounter, recognize, enter, and modulate cells. Four interrelated determinants are considered: lipid fluidity and domain organization; membrane mechanics and deformability; electrochemical properties and ion channel regulation; and glycocalyx-mediated immune recognition. Together, they shape ligand–receptor binding, adhesion, endocytosis, trafficking, immune recognition, and downstream delivery, providing a mechanistic basis for interpreting disease-associated remodeling, membrane-guided strategies, and translational qualification.

Membrane interactions also have kinetic dimensions not captured by equilibrium affinity or endpoint uptake. Association and dissociation rates, receptor-binding residence time, and collective adhesion timescales influence whether engagement is transient or persistent and whether it supports sustained receptor modulation, sequestration, or kinetically trapped membrane-bound states. Multivalent systems illustrate why stronger or less reversible binding is not uniformly advantageous. Increased ligand valency can prolong receptor residence and, together with ligand spacing, alter downstream signaling [46], whereas strong multivalent nanoparticle adhesion in a model membrane system can rapidly sequester available receptors and generate long-lived out-of-equilibrium membrane-bound states [47]. Kinetic parameters should therefore be matched to the intended mechanism rather than uniformly optimized for maximal affinity, prolonged residence, or rapid uptake. Table 1 summarizes these determinants, their linked events, design implications, and principal boundaries.

### 3.1. Membrane Fluidity, Lipid Phase Behavior, and Domain Organization

Membrane fluidity and phase behavior arise from the coupled organization of lipids and proteins. Lipid composition, fatty acid unsaturation, phase state, and sterol balance regulate lateral mobility, membrane order, permeability, and membrane protein nanoenvironments [12,14,40,57,58]. Remodeling is context-dependent: oxidative stress increased rigid membrane domains in THP-1-derived macrophages [59], whereas altered red blood cell (RBC) membrane fluidity has been reported in diabetes [60]. Lipid–protein coupling regulates membrane protein mobility, organization, and activity [11], while dynamic rafts and nanodomains can concentrate or exclude receptors and signaling components [15,22]. Receptor function therefore depends on local lipid organization as well as receptor abundance [40].

Fluidity and phase state also affect carrier engagement and entry. In coarse-grained molecular dynamics simulations of a lipid vesicle–planar membrane system, cholesterol modulated wrapping and fusion in a composition-dependent manner [61]. Lipid-dependent changes in packing, permeability, and dynamics [57,58] can alter interaction energetics [17] and may influence endocytic efficiency and route selection [42]. Membrane association, however, is not a binary transition from adsorption to internalization. In supported lipid bilayers, surface-adsorbed CdTe quantum dots produced charge-dependent perturbations extending from the exposed leaflet into the hydrophobic region and, with increasing positive membrane charge, toward the inner leaflet, accompanied by increased hydration, defects, and reduced nanomechanical stability [62]. Nanoscale localization is therefore a context-dependent characterization parameter that distinguishes particle position from the depth and extent of membrane perturbation, not an additional intrinsic membrane determinant. Excessive endosomal perturbation can also activate damage-sensing inflammatory responses, as demonstrated for LNP endosomal escape [45].

For lipid-based nanomedicines, bilayer phase behavior can influence permeability, carrier stability, and drug release [58,63]. The Collective Small Displacements model illustrates how equilibrium properties can be linked quantitatively to transport kinetics: using the thermal expansion coefficient of lipid vesicles as a single experimentally accessible input, it predicted lipid-exchange activation energies and transfer timescales without adjustable parameters and was validated by label-free quartz crystal microbalance with dissipation monitoring (QCM-D) across multiple lipid systems [64]. Although developed for lipid exchange in model membranes rather than nanocarrier delivery, this work provides proof of principle for converting an equilibrium property into predictive transport parameters. This framing suggests a trade-off between circulation stability and local release, while delivery after endocytic uptake remains constrained by endosomal escape [63]. Lipid-state variables are therefore design-relevant when they alter receptor organization, membrane association, uptake route, or release rather than when measured only as standalone descriptors.

### 3.2. Membrane Mechanics, Stiffness, and Deformability

Membrane-associated mechanics include plasma membrane tension and membrane–cortex coupling as well as cell-scale stiffness, elastic modulus, and deformability, which reflect membrane–cytoskeletal organization rather than membrane properties alone [16,48,65,66]. Membrane tension is dynamically set by membrane–cortex attachment and cytoskeletal forces [16] and, in theoretical nanoparticle–membrane models, alters the energetic cost of wrapping and the threshold for adhesion-driven uptake [17]. Through feedback with cytoskeletal remodeling and membrane trafficking [66,67], plasma membrane tension coordinates phagocytosis [65] and helps maintain leading-edge polarity during directed migration [68,69], linking local mechanics to wrapping, phagocytosis, and migration.

Carrier mechanics and target-cell mechanics are distinct but interacting variables that can influence deformation and membrane wrapping [17,48,49]. In a direct preclinical comparison, Kong et al. showed that changing the stiffness of otherwise compositionally matched layer-by-layer nanoparticles altered elimination half-life, tumor accumulation, and intratumoral penetration [23]. Disease-associated mechanical remodeling is likewise context-dependent. Atomic force microscopy (AFM) showed that patient-derived metastatic cancer cells were substantially less stiff than benign reactive mesothelial cells [70], whereas broader analyses indicate variation across cancer types, cellular states, and microenvironments [71]. In vascular disease, disturbed flow is a hemodynamic context modifier of endothelial mechanosensing rather than an intrinsic membrane property [72]. Membrane and carrier mechanics are design-relevant when linked to wrapping, phagocytic processing, circulation, accumulation, or tissue penetration, not when treated as isolated scalar descriptors.

### 3.3. Electrical Properties, Charge Distribution, and Ion Channel Regulation

Membrane potential and ion channel activity define an electrochemical context linking excitability, disease-associated signaling, and therapeutic intervention [41]. Channel dysfunction contributes to altered neuronal excitability [31], cardiovascular pathophysiology [73], and defective epithelial ion and fluid transport in cystic fibrosis [74]. An artificial chloride channel disrupted intracellular chloride homeostasis and induced cancer cell apoptosis through autophagy perturbation, demonstrating the functional consequences of channel-level intervention [75]. Ion channels are therefore membrane-embedded targets through which pharmacological or engineered modulators can alter ion flux, excitability, and downstream responses [41,76]. In membrane-guided nanomedicine, however, channel activity and membrane potential constitute a local electrochemical context; they do not by themselves establish selective nanocarrier targeting or delivery.

Nanoparticle surface charge, assessed by zeta potential under defined medium conditions, can modulate membrane association and uptake, but these effects depend on surface chemistry, biological medium, and protein corona formation and are not inherently selective [50]. The corona is an acquired nanocarrier property rather than an intrinsic cell membrane property and can reshape biological identity and subsequent nanoparticle–cell interactions [56].

Electrochemical impedance spectroscopy (EIS) provides a complementary label-free readout of interfacial binding and cell-associated impedance changes [51,77]. In cell-based systems, EIS can report membrane integrity, barrier properties, and signaling-associated responses, but these readouts remain indirect and context-dependent [51]. Electrical variables are design-relevant when they alter channel function, membrane association, or uptake in the relevant biological medium; zeta potential alone does not demonstrate disease-selective delivery [50].

### 3.4. Glycocalyx, Membrane Proteins, and Immune Recognition

The glycocalyx and membrane proteins form a surface-recognition layer that shapes membrane organization and immune recognition [10]. Glycosylation patterns and surface glycoconjugates contribute to context-dependent self, altered-self, and non-self recognition [52,53]. Park et al. showed that cancer-associated mucins and their glycosylation contribute to nanoscale glycocalyx thickness and that natural killer cell-mediated cytotoxicity decreases as glycocalyx thickness increases [78]. At the nano–bio interface, material-intrinsic surface properties can affect immune activation, suppression, or clearance [44], whereas acquired biomolecular coronas can reshape biological identity and immune recognition [56]. Cell-surface and endosomal Toll-like receptors further convert molecular recognition into cytokine and interferon signaling [54].

For nanomedicine design, glycocalyx-associated motifs and membrane protein identity are relevant only when their accessibility is established in the intended biological context and mechanistically linked to binding, uptake, immune modulation, or downstream delivery. Polyethylene glycol (PEG)-functionalized nanoparticles illustrate that surface modification can alter protein adsorption and cellular uptake [24], but these effects cannot be inferred from nominal surface chemistry alone. Membrane-derived motifs may provide source-dependent recognition cues [27,55], but their therapeutic value requires exposure and functional coupling to the intended interaction. Glycocalyx-associated glycans and membrane proteins are therefore treated as determinants of immune recognition and membrane compatibility, not by themselves as disease-specific targets or qualified platform functions.

## 4. Disease-Associated Membrane Remodeling: Therapeutic Entry Points and Delivery Barriers

Disease-associated membrane remodeling links the biophysical determinants in Section 3 to the strategies in Section 5 but should not be treated as a catalog of disease targets. Across cancer, neurodegeneration, inflammation, infection, and vascular disease, remodeling affects lipid organization, mechanical state, functional membrane nodes, and glycocalyx-associated immune recognition. These changes may create candidate entry points by altering recognition, adhesion, uptake, immune engagement, or membrane vulnerability, but their exploitation is limited by membrane heterogeneity and accessibility and by contextual barriers such as tissue architecture, immune recognition, hemodynamics, and disease stage. A remodeled feature is treated as a candidate entry point only when it is accessible, disease-biased, and functionally linked to recognition, uptake, retention, immune engagement, or transport across the relevant biological barrier. Platform-specific implementation is considered in Section 5.

Because the evidence spans experimental levels, we distinguish three types of inference: experimentally demonstrated mechanisms, supported by perturbation or intervention studies that establish a functional link; associative evidence, which identifies correlations with disease state or phenotype without establishing causality; and inferred design opportunities, which propose candidate nanomedicine entry points without direct demonstration of selective delivery. Evidence is also classified by model type, including model membranes, cultured cells, organoids, animal models, and human specimens or clinical studies. Table 2 links each disease context to its dominant membrane alterations, functional consequences, candidate entry points, delivery boundaries, and representative evidence.

### 4.1. Altered Membrane Fluidity and Lipid Metabolism

Altered membrane fluidity and lipid metabolism are common forms of disease-associated remodeling. Changes in lipid composition, cholesterol balance, lipid order, and membrane dynamics can reshape receptor signaling, trafficking, immune interactions, and drug responsiveness and may connect disease-associated composition to therapeutic access.

#### 4.1.1. Shared Lipid Fluidity Remodeling Across Diseases

Across diseases, lipid fluidity remodeling is associated with signaling and therapeutic interactions, but evidence varies by disease and model. Membrane physical state shapes membrane protein nanoenvironments and can modulate protein function, ion channel behavior, and transporter activity [40,101]; its determinants include lipid composition, sterol content, leaflet asymmetry, fatty acid saturation, and lipid–protein coupling [14,102]. Healthy mammalian plasma membranes show leaflet-specific differences in lipid unsaturation, packing, and diffusivity [36], suggesting that disease remodeling may affect transbilayer lipid distribution or leaflet-specific states as well as bulk composition. Such claims require leaflet-resolved measurements because bulk lipidomics and whole-membrane fluidity measurements do not establish altered leaflet asymmetry.

Human studies report altered RBC membrane fluidity in diabetes [60], whereas oxidative stress experimentally increased rigid membrane domains in cultured THP-1-derived macrophages [59]. Tissue lipid remodeling has also been reported in metabolic disease and Alzheimer’s disease (AD) [84,103], but does not alone establish altered plasma membrane fluidity or causal effects on nanocarrier delivery. Design relevance therefore requires disease enrichment, accessibility, functional linkage to uptake or signaling, and distinction from physiological variability.

#### 4.1.2. Cancer: Lipid-State Remodeling, Invasion, and Drug Response

In cancer, lipid metabolic remodeling is associated with progression, invasion, and metastasis [79], while altered fluidity has been linked to phenotype and treatment response across heterogeneous evidence levels [104]. In a multiscale comparison, highly aggressive PC-3 prostate cells were less stiff and viscous than non-tumoral WPMY-1 cells, and monolayers prepared from PC-3 lipids showed faster fluorescence recovery after photobleaching (FRAP)-measured lateral diffusion [105]. This provides associative cultured-cell and extracted-lipid-monolayer evidence, not proof that fluidity causally drives metastasis. Reviews indicate that perturbing cholesterol-rich domains or raft-associated proteins can alter receptor organization, internalization, and survival or death signaling [106,107].

Direct evidence for fluidity-guided carrier–membrane interactions remains limited. In cultured cancer cells, Bompard et al. showed that tuning liposome fluidity enabled selective engagement of cell lines with different aggressiveness and supported carrier–cell membrane fusion [108]. This establishes membrane fluidity as an in vitro carrier-design variable, not endogenous tumor fluidity as a universal in vivo delivery determinant. Preclinical studies also support membrane-lipid perturbation as a treatment strategy [109], which is distinct from fluidity-based targeting. Interpretation must therefore account for tumor type, metabolic and stromal context, epithelial–mesenchymal state, and treatment pressure.

#### 4.1.3. Alzheimer’s Disease: Neuronal Lipid Dysregulation and Amyloid-β–Membrane Injury

Human brain lipid analyses provide evidence of AD-associated remodeling. Han et al. identified substantial sulfatide deficiency and ceramide elevation in very early AD [82], while lipidomic and genetic evidence supports disrupted cholesterol and sphingolipid homeostasis [84]. These findings establish brain lipid remodeling, not altered plasma membrane fluidity or a causal relationship with nanocarrier delivery.

At the interaction level, all-atom molecular dynamics simulations showed that a neuronal membrane-mimicking lipid environment alters amyloid-β42 (Aβ42) dimer conformations, with GM1 sugar groups as major interaction partners [83]. This complements broader model and cellular evidence linking amyloid-β (Aβ) biology to cholesterol-rich membrane environments and domain organization [85]. Aβ-associated dysfunction can also involve disturbed calcium homeostasis, oxidative stress, and synaptic injury [110]. These findings support Aβ–membrane interactions as a mechanistic vulnerability, whereas broader AD lipid remodeling remains partly associative.

Direct evidence that these lipid states improve nanocarrier delivery remains limited. The BBB is a separate barrier-level requirement, and trans-BBB transport does not establish adequate parenchymal distribution or neuronal membrane engagement [87,88]. Neuroinflammation imposes an additional constraint [86]. AD-associated lipid remodeling should therefore remain a candidate design cue unless directly linked to Aβ–membrane interactions, neuronal uptake, or treatment response.

#### 4.1.4. Atherosclerosis: Plaque Lipid Signatures and Vascular Membrane Remodeling

In atherosclerosis, lipid remodeling spans plaque- and cell membrane-level processes. Spatial metabolomics of human carotid plaques identified region- and stage-dependent cholesterol-, phospholipid-, and sphingolipid-related signatures [97]. This provides human tissue-level associative evidence of plaque heterogeneity, not proof of plasma membrane remodeling or a causal delivery determinant. At the cellular level, cholesterol- and sphingolipid-rich domains contribute to receptor organization and inflammatory signaling, as summarized in a lipid raft review [111]. Primary studies identified persistent inflammarafts—enlarged cholesterol-rich membrane domains containing inflammatory receptor complexes—in macrophage models and murine atherosclerotic lesions [112], with broader implications reviewed elsewhere [113]. Macrophage metabolic and functional states also vary with plaque microenvironment and disease stage in human and experimental atherosclerosis [114].

These observations establish the mechanistic relevance of membrane domain and macrophage-state remodeling, not validated nanocarrier-delivery determinants. Plaque lipid states, inflammarafts, and macrophage phenotypes therefore require direct evidence of carrier accessibility, binding, retention, uptake, or release under relevant vascular conditions. Delivery also depends on endothelial access, blood flow, lesion stage, and blood-contact compatibility; lipid remodeling is most informative when localized to an accessible interface and functionally linked to delivery or treatment response.

### 4.2. Membrane-Associated Mechanical Remodeling in Disease

Membrane-associated mechanical remodeling includes plasma membrane tension, membrane–cortex coupling, cytoskeletal force transmission, and cell-surface deformability. These properties are distinguished from ECM stiffness, stromal confinement, and tissue-scale mechanics, which are contextual factors relevant when they modulate membrane mechanosensing, carrier access, or membrane–therapeutic interactions. Membrane-associated and contextual mechanics can influence migration, adhesion, phagocytosis, shear sensing, carrier interactions, and tissue transport, but their delivery relevance requires functional linkage to uptake, retention, or transport beyond physiological variability. Cancer, inflammation, and vascular disease illustrate both candidate opportunities and mechanical barriers.

#### 4.2.1. Shared Mechanical Remodeling Across Diseased Cells and Tissues

Membrane tension, membrane–cortex coupling, and cytoskeletal forces regulate cell-surface mechanics, cell shape, and responses to mechanical or biochemical stimuli [16]. In disease, altered stiffness, elastic modulus, and deformability are associated with cell–matrix and cell–cell interactions, particularly in cancer and inflammation [30]. More broadly, mechanobiology links cellular force sensing and generation to tissue form and function in development and disease [115].

Nanomedicine relevance depends on the level at which mechanics is defined. Theoretical models predict that particle and membrane properties influence nanoparticle adsorption and membrane deformation [17], whereas particle–endothelium interactions under flow additionally depend on hemodynamics, particle properties, and endothelial activity [100]. Preclinical experiments show that carrier stiffness can alter pharmacokinetics, tumor accumulation, and penetration [23]. Because carrier stiffness is an engineered property, these findings do not establish diseased-cell stiffness as a delivery determinant. Disease-associated membrane or cell-surface mechanics remains a candidate cue unless an accessible feature is directly linked to carrier interaction, uptake, retention, or transport.

#### 4.2.2. Cancer Invasion: Deformability, ECM Mechanics, and Mechanotransduction

In cancer, delivery depends on the interplay among tumor-cell deformability, ECM mechanics, and tissue-level transport barriers. AFM measurements of patient-derived metastatic cancer cells from body-cavity fluids showed substantially lower stiffness than benign reactive mesothelial cells, providing human cell-level associative evidence [70]. Increased matrix stiffness can enhance integrin-dependent adhesion and malignant behavior [116], while collagen crosslinking increases ECM stiffness, activates focal adhesion and integrin–phosphoinositide 3-kinase signaling, and promotes invasion in experimental models [117]. Yes-associated protein (YAP) and transcriptional coactivator with PDZ-binding motif (TAZ) are experimentally identified nuclear mediators of ECM rigidity and cell-shape cues [118], although the effects of ECM stiffness, architecture, and confinement remain context-dependent [119,120].

Disease-associated mechanics must be distinguished from engineered carrier mechanics. Compliant layer-by-layer nanoparticles showed prolonged elimination, increased tumor accumulation, and deeper penetration while retaining the same outer targeting chemistry [23]. Soft tumor-cell-derived microparticles also enhanced tumor accumulation, extravasation, and parenchymal penetration in tumor-bearing mice [121]. These findings establish carrier mechanics as a preclinical design parameter but do not show that reduced tumor-cell stiffness improves delivery. ECM architecture, stromal confinement, and mechanical heterogeneity further diversify the tumor microenvironment [120], while ECM transport barriers and interstitial pressure restrict tissue-level delivery [122]. These factors can uncouple cell-level mechanical phenotypes from effective tumor delivery.

#### 4.2.3. Inflammation: Membrane Tension, Immune Cell Mechanosensing, and Phagocytosis

Cell-level studies show that plasma membrane tension coordinates trafficking, cytoskeletal remodeling, and signaling during phagocytosis and contributes to neutrophil polarity [65,69]. At the tissue level, ECM mechanics and other physical cues regulate immune-cell positioning, migration, and activation through mechanotransduction [123], while ECM remodeling interacts reciprocally with immune-cell recruitment and function during inflammation and repair [124]. Human fibrotic lung analyses and engineered macrophage–fibroblast microtissues support mechanical activation of profibrotic macrophages and implicate integrin αMβ2–ROCK2 signaling [125]. Mechanical deformation of primary human neutrophils induced rapid loss of polarity in a microcirculation-mimetic system [126], whereas stiffer substrates increased inflammation-associated tension and permeability in an in vitro endothelial monolayer model [127].

These findings establish mechanosensitive immune and endothelial responses, not selective nanocarrier-delivery determinants. Deformable immune cells, mechanically stressed endothelium, and fibrotic matrices remain candidate interfaces requiring direct linkage to carrier adhesion, phagocytic uptake, retention, endothelial passage, or tissue transport. Their relevance is stage- and context-dependent: inflammatory endothelial activation can increase permeability [127], whereas chronic fibrotic remodeling can impose matrix stiffening and transport barriers [124,125]. The relevant mechanical state must therefore be accessible and linked to the intended delivery process.

#### 4.2.4. Vascular Stiffness: Endothelial Mechanics, Shear Stress, and Delivery Barriers

Vascular-cell mechanosensing occurs within broader hemodynamic and tissue-mechanical contexts. Shear stress and pressure are sensed through endothelial and vascular smooth-muscle mechanotransduction pathways [128,129], whereas ECM stiffness is a vessel-wall property that can reshape vascular-cell mechanosensing and function [130]. Neither is an intrinsic membrane biophysical property. In pulmonary hypertension, stiff ECM activates YAP/TAZ-dependent metabolic and proliferative remodeling in cultured pulmonary vascular cells, with intervention-based validation in rats and correlative evidence from nonhuman primates and humans [131].

Vascular mechanics likewise require evidence-level distinction in nanomedicine. Pathological high shear can serve as a delivery trigger: shear-activated nanoparticle aggregates disassembled under high-shear conditions and enhanced thrombolytic activity in mouse models of vascular occlusion [98]. This is hemodynamic rather than membrane-specific targeting. More generally, particle–endothelium interactions under flow depend on hemodynamics, blood constituents, particle properties, and endothelial state [100]. Blood-contact safety is a separate constraint because complement-dependent opsonization and immune uptake vary across nanomedicine types, donors, and species [99]. Arterial stiffness is therefore an integrative vessel-wall and hemodynamic phenotype shaped by ECM remodeling, vascular-cell state, inflammation, endothelial dysfunction, and blood pressure, not an intrinsic membrane property [132].

### 4.3. Functional Remodeling of Membrane Receptors, Ion Channels, and Signaling Domains

Beyond lipid and mechanical remodeling, receptors, ion channels, and signaling domains govern extracellular sensing, signal transduction, ion flux, and pathogen entry. Their alteration in cancer, channelopathies, and infection can contribute to disease and create candidate therapeutic entry points. Design relevance requires surface accessibility, functional linkage to signaling, uptake, or pathogen entry, and sufficient distinction from physiological expression in healthy tissues.

#### 4.3.1. Receptors, Channels, and Signaling Domains as Functional Remodeling Nodes

Membrane receptors, ion channels, and transporters mediate sensing, signal transduction, ion flux, and molecular transport, and many are established pharmacological targets [38]. Disease relevance, however, is not determined by expression alone. Lateral organization into lipid rafts and other domains can concentrate or segregate receptors and signaling proteins, shaping clustering, activation, and downstream signaling [15,21]. Glycosylation further regulates protein folding, trafficking, stability, and surface presentation [52]. Candidate cues must therefore be evaluated through membrane localization, domain organization, glycosylation, trafficking dynamics, and disease-linked function as well as abundance.

#### 4.3.2. Tumor Receptors: Dysregulated Recognition, Endocytosis, and Targeting Boundaries

In cancer, receptor dysregulation spans distinct evidence levels. Reviews indicate that mutation, amplification, or overexpression of receptor tyrosine kinases can sustain oncogenic signaling and therapeutic dependence in selected contexts [80]. A systems-level primary analysis identified rewired G protein-coupled receptor (GPCR)–ligand and GPCR–biosynthetic enzyme axes associated with cancer subtype and survival; selected GPCR-directed drugs showed anti-growth associations in large-scale cancer cell screens, with two candidates further validated experimentally [133]. These findings establish disease relevance and, for selected receptors, pharmacological actionability, not nanocarrier targeting.

Receptor-mediated delivery depends on ligand valency, internalization, and endocytic trafficking, which influence nanoparticle uptake and intracellular processing [134]. Preclinical literature supports ligand–receptor targeting, but tumor heterogeneity and tissue transport barriers can limit delivery [135]. Elevated expression therefore remains a candidate cue: receptor-guided uptake requires accessible binding and internalization, whereas productive intracellular delivery additionally requires appropriate trafficking and cargo release.

#### 4.3.3. Ion Channel Remodeling and Disease-Associated Bioelectricity

Membrane potential and ion channel activity regulate ion flux, excitability, electrical signaling, and cellular homeostasis [41]. Transient receptor potential channels illustrate how channels convert chemical, thermal, mechanical, and osmotic cues into downstream responses [136]. Channelopathy reviews summarize human genetic and clinical evidence linking pathogenic channel variants to neurological, cardiovascular, and other disorders [137]. Cystic fibrosis provides a causal example: a foundational study established the cystic fibrosis transmembrane conductance regulator (CFTR) as a cyclic adenosine monophosphate-regulated chloride channel [138], while subsequent genetic, mechanistic, and clinical evidence showed how pathogenic *CFTR* variants disrupt epithelial ion and fluid transport [74]. These findings establish channel dysfunction as a disease mechanism in defined settings, not channel state or membrane potential as a determinant of selective nanocarrier delivery.

Pharmacological modulation of ion channels or bioelectric state must therefore be distinguished from nanocarrier targeting. Many channels are broadly distributed and participate in interconnected signaling networks, making tissue context and off-target electrophysiological effects important for systemic modulation [41,136]. Disease-associated channel activity or membrane potential remains a candidate functional cue unless direct evidence demonstrates disease-selective carrier engagement or delivery through that feature. Without such evidence, channel remodeling supports local pharmacological or bioelectric modulation rather than selective membrane targeting.

#### 4.3.4. Infectious Entry Receptors: Receptor Engagement, Protease Activation, and Membrane Fusion

In infection, host receptors and entry-associated proteases are key determinants of pathogen entry. Severe acute respiratory syndrome coronavirus 2 (SARS-CoV-2) engages angiotensin-converting enzyme 2 (ACE2), while proteolytic spike activation enables cell-surface or endosomal routes that ultimately require viral–cell membrane fusion [95]. In differentiated human airway epithelium, SARS-CoV-2 preferentially replicates in ciliated cells and is released apically [93]. Human nasal tissue and early coronavirus disease 2019 analyses further link viral tropism to multiciliated epithelial cells and apical localization of entry factors [139]. Clustered regularly interspaced short palindromic repeats (CRISPR)/CRISPR-associated protein 9 (Cas9) loss-of-function experiments in human intestinal organoids showed that ACE2 is the obligate entry receptor and that transmembrane serine protease 2 is required for efficient viral replication and spread in this model [94].

Productive infection therefore depends on receptor localization, protease availability, cell type, tissue tropism, and entry pathway, not receptor expression alone. A mechanistically required host receptor or protease may support entry blockade but does not establish selective nanocarrier delivery. Infectious entry factors remain candidate membrane-guided cues only when their accessibility, tissue or cell-type distribution, and selectivity for the intended context are established and linked to the proposed mechanism.

### 4.4. Glycosylation and Disease-Associated Immune Recognition Phenotypes

Beyond receptors, ion channels, and pathogen-entry nodes, disease-remodeled membranes present a glycan-rich surface layer that contributes to immune recognition. Tumors can exhibit aberrant glycosylation and glycocalyx thickening, whereas inflammation can remodel endothelial and immune-cell glycosylation to influence leukocyte recruitment, vascular adhesion, and signaling. These features are candidate design cues only when disease-enriched, accessible to immune cells or carriers, functionally linked to immune modulation, adhesion, uptake, or retention, and sufficiently stable in the relevant context.

#### 4.4.1. Glycocalyx and Membrane Proteins as a Self and Non-Self Recognition Layer

Cell-surface glycans and membrane proteins form a recognition layer rather than passive structural decoration. Glycosylation regulates membrane protein folding, trafficking, stability, surface presentation, and function, and activity may consequently depend on glycosylation state rather than protein abundance alone [52]. At the immune interface, glycans can encode self-associated molecular patterns, while glycan-binding proteins decode these structures to regulate tolerance and activation [53]. Glycocalyx architecture and molecular crowding further influence receptor accessibility, organization, and ligand interactions [10,140].

#### 4.4.2. Cancer Immune Evasion

In cancer, glycosylation remodeling can promote immune evasion and steric protection at the cell surface. Reviews frame aberrant tumor glycosylation as a network of glyco-immune checkpoints rather than passive decoration [81]. Tumor-associated sialoglycans can engage inhibitory sialic acid-binding immunoglobulin-like lectins (Siglecs) on immune cells and suppress antitumor responses [141]. Direct experiments further showed that cancer-associated mucins and their glycosylation increase nanoscale glycocalyx thickness, which is associated with reduced susceptibility to cytotoxic immune-cell attack; enhanced receptor-mediated activation and effector-cell display of glycocalyx-editing enzymes improved cytotoxicity against mucin-bearing cells [78].

Additional mechanisms include N-glycosylation of the programmed cell death protein 1 (PD-1)–programmed death-ligand 1 (PD-L1) axis, which can regulate checkpoint stability, surface expression, and responses to checkpoint-directed interventions [142], and galectin–glycan interactions implicated in immune exclusion and tumor progression [143]. These findings establish tumor glycosylation as an immune-regulatory membrane phenotype, not glycan abundance as an actionable delivery determinant.

#### 4.4.3. Inflammatory Glycocalyx Remodeling and Recognition

In inflammation, the glycocalyx and glycosylation landscape undergo active remodeling as well as damage. Endothelial glycocalyx turnover and shedding can contribute to barrier dysfunction, altered permeability, and inflammatory endothelial dysfunction [90]. A recent review links endothelial glycosylation to surface recognition and signaling in pulmonary vascular disease while emphasizing that its mechanistic and therapeutic significance remains incompletely defined [144].

Selectin-dependent recognition of sialylated leukocyte ligands provides an established inflammatory adhesion mechanism. In human leukocytes, disruption of ST3 β-galactoside α-2,3-sialyltransferase 4 markedly reduced selectin-dependent tethering and rolling [89], while sialyl Lewis X-containing ligands participate in E-selectin-mediated recruitment on activated endothelium [91]. Inflamed endothelium therefore represents a candidate nanomedicine recognition interface [91], but carrier adhesion and retention require validation under relevant blood-flow conditions [100].

Inflammation-associated N-glycosylation can also modulate immune-cell effector functions, cellular interactions, and signaling [145]. Myeloid C-type lectin receptors participate in innate recognition, phagocytosis, cytokine production, and antigen presentation [146], whereas Siglec–sialoglycan interactions can regulate macrophage phenotype and inflammatory function [147]. These mechanisms establish a dynamic recognition system, not selective delivery determinants. Candidate cues therefore require disease enrichment, vascular-bed and disease-stage specificity, temporal stability, and direct linkage to carrier adhesion, uptake, retention, or transport.

#### 4.4.4. Disease-Associated Recognition Codes as Candidate Design Templates

Disease-associated recognition codes are composite membrane signatures formed by proteins, glycans, lipids, and immune recognition motifs that vary with disease and cell state. Their design value depends on disease enrichment, surface accessibility, spatial organization, and functional coupling to binding, adhesion, uptake, immune modulation, or retention; they should not be treated as actionable targets by default. Such claims require combined molecular and spatial evidence rather than abundance measurements alone.

Even attractive molecular features require validation across relevant biological barriers because successful recognition does not ensure adequate target-site accumulation or effective delivery [4]. For cell membrane-coated systems, preservation and characterization of relevant membrane components and functions during fabrication must be demonstrated rather than assumed [148]. The remodeling features described in Section 4 therefore define candidate opportunities whose therapeutic value must be established at the strategy level, not inferred from descriptive remodeling alone.

## 5. Membrane-Guided Nanomedicine Strategies: Targeting, Engineering, and Disease-Context Matching

### 5.1. From Disease-Altered Membranes to Therapeutic Entry Points

Disease-altered membrane features are candidate therapeutic interfaces, not automatically exploitable delivery targets. Here, a therapeutic interface is a remodeled feature whose accessibility or functional state may support recognition, adhesion, uptake, immune modulation, local pharmacological action, or controlled release. Receptors, lipid domains, glycocalyx structures, ion channel and bioelectric states, and membrane-associated mechanics operate at different mechanistic levels. Surface features may support ligand- or motif-dependent engagement, whereas channel and membrane potential states more often support functional modulation unless disease-selective delivery is directly demonstrated. This section therefore matches membrane features with intervention mechanisms, engineered platforms, disease contexts, and testable design boundaries.

**Figure 2:**
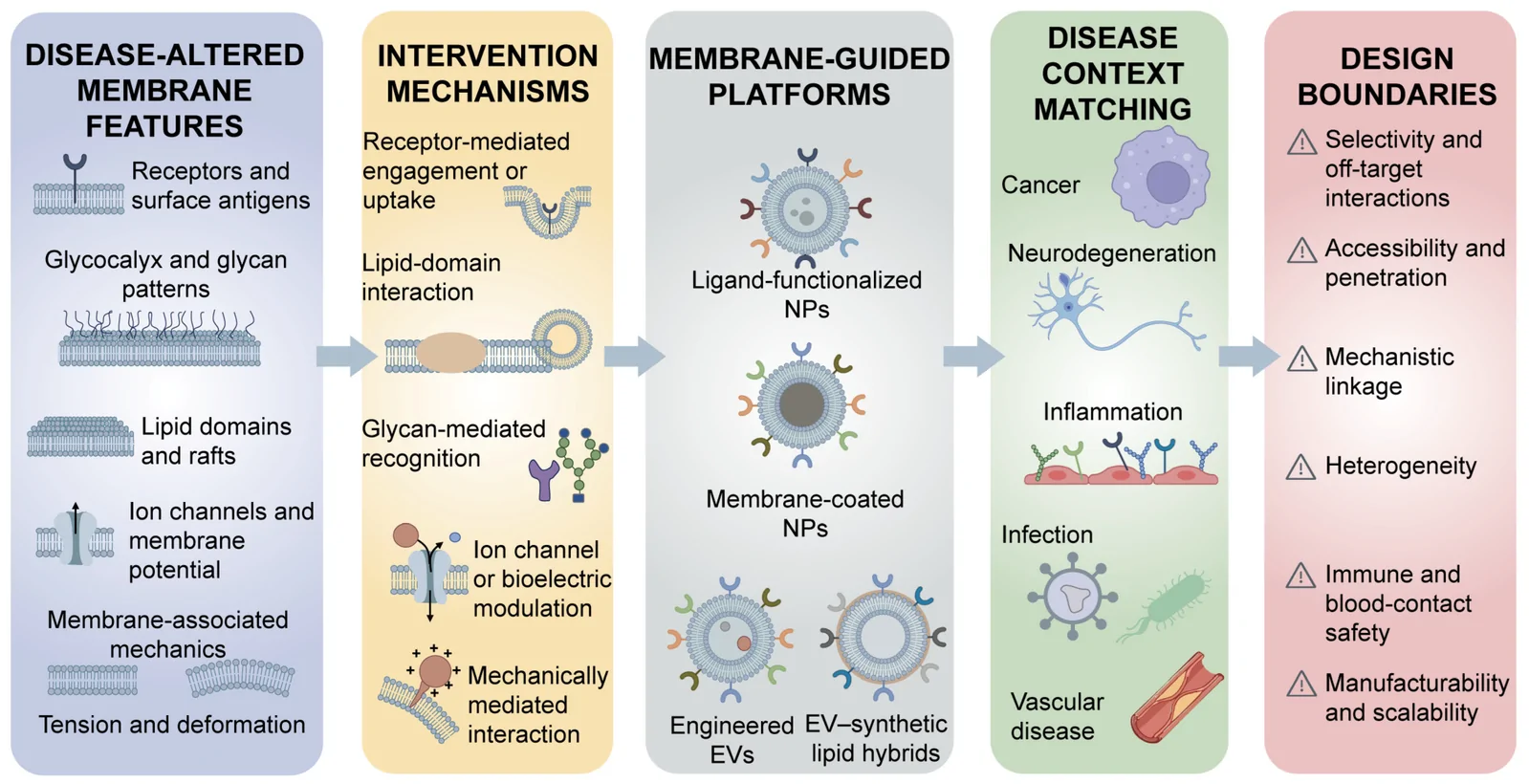
Context-matched design of membrane-guided nanomedicine strategies. This schematic links candidate disease-altered membrane features to intervention mechanisms and representative platform classes, which are then matched to disease contexts and evaluated against defined design boundaries. Candidate features include receptors and surface antigens, glycocalyx and glycan patterns, lipid domains and rafts, ion channel activity and membrane potential, and membrane-associated mechanics. Intervention mechanisms encompass receptor-mediated engagement or uptake, lipid-domain interaction, glycan-mediated recognition, ion channel or bioelectric modulation, and mechanically mediated membrane interaction. Representative platforms include ligand-functionalized nanoparticles, membrane-coated nanoparticles, engineered extracellular vesicles, and EV–synthetic lipid hybrids. Strategy relevance depends on selectivity and off-target interactions, accessibility and penetration, mechanistic linkage, heterogeneity, immune and blood-contact safety, manufacturability, and scalability. Platform identity alone does not define a membrane-guided strategy. “+” denotes positive surface charge. EVs, extracellular vesicles; NPs, nanoparticles.

Membrane-guided platforms also have distinct architectures. Ligand-functionalized nanoparticles display defined ligands on engineered surfaces, whereas membrane-coated nanoparticles transfer shells derived from RBCs, platelets, immune cells, cancer cells, or hybrid membranes onto synthetic or semisynthetic cores. EV-based systems comprise cell-released vesicles with membrane-associated and luminal components and can undergo surface or cargo engineering. EV–liposome and EV–LNP hybrids combine EV-derived components with synthetic lipid modules. Figure 2 outlines these architectures, while Table 3 compares their mechanisms, representative evidence, and principal design boundaries.

Accessible disease-altered receptors and other surface-recognition nodes provide the most direct delivery entry points. In cancer, dysregulated receptor networks establish disease-relevant surface biology [80], but delivery requires receptor accessibility, internalization, and appropriate trafficking [134]. In inflammation, cell membrane-coated platforms can transfer adhesion or decoy functions relevant to activated immune and endothelial interfaces [149]. Neutrophil membrane-coated nanoparticles provide direct preclinical evidence of proinflammatory cytokine neutralization and therapeutic efficacy in arthritis models [150]. In infection, host entry receptors can support decoy or entry-blockade strategies when mechanistically linked to pathogen entry [95]. ACE2-rich membrane nanoparticles directly inhibited SARS-CoV-2 entry and infection in vitro and in vivo [151]. Receptor-based strategies therefore require accessible and context-selective targets coupled to the intended internalization or blockade mechanism.

By contrast, ion channels and membrane potential primarily support functional modulation. Channel dysfunction contributes to channelopathies [137], and review-level evidence links ion channel activity and bioelectric modulation to cancer phenotypes and pharmacological strategies [152,153], but not selective nanocarrier targeting. Without direct evidence of disease-selective carrier engagement or delivery through these states, they are better treated as opportunities for local pharmacological or bioelectric modulation. Lipid domains and rafts organize receptors and signaling proteins [15] and can influence endocytic routing [154], while glycosylation and glycocalyx remodeling shape protein function and immune recognition [52,53]. Tumor glyco-immune remodeling [81] and endothelial glycocalyx turnover [90] therefore identify disease-associated surface contexts whose delivery relevance requires context-specific validation.

Membrane mechanics and carrier physicochemical properties are distinct but interacting design levels. Membrane tension regulates cell-surface mechanics and phagocytic processing [16,65], whereas disease-associated mechanics in cancer and inflammation impose contextual constraints [30]. At the carrier level, theoretical modeling predicts elasticity-dependent differences in membrane wrapping and uptake [49], while preclinical studies show that stiffness can influence circulation, tumor accumulation, and penetration [23]. Surface chemistry and charge can also affect protein adsorption, membrane association, and cellular uptake [24,50]. These carrier properties are context-dependent engineering parameters, not intrinsic disease-membrane targeting features.

The following sections distinguish selective engagement, functional modulation, and biomimetic or engineered membrane strategies and evaluate each against disease enrichment, accessibility, barrier context, and safety.

### 5.2. Molecular and Biophysical Mechanisms of Membrane Engagement and Intervention

Membrane-guided intervention is a multistep process in which recognition, adhesion, uptake, trafficking, tissue penetration, functional modulation, and immune compatibility depend jointly on membrane organization, carrier design, and biological context. A ligand, membrane coating, or physicochemical modification is useful only when coupled to an accessible membrane feature and a productive outcome, such as selective engagement, uptake, signaling modulation, immune redirection, or local release. The required temporal profile is mechanism dependent: surface-confined functions may require persistent association, whereas intracellular delivery requires timely progression from binding to internalization and, where necessary, cargo release. Association and dissociation kinetics, interaction lifetime, and residence time should therefore be matched to the intervention rather than uniformly optimized for maximal affinity, persistence, or uptake [46,47].

Five intervention classes are considered: receptor-mediated engagement and uptake; lipid-domain-associated interaction and uptake; glycan-mediated recognition; ion channel and bioelectric modulation; and mechanically mediated membrane interaction. Receptor and glycan cues may support molecular recognition, channels and bioelectric states primarily support functional modulation, lipid domains influence receptor organization and uptake routes, and mechanical interactions depend jointly on membrane and carrier properties. Design should therefore begin with a mechanistically defined disease-associated feature and intervention mode rather than an isolated ligand or broadly defined membrane phenotype.

#### 5.2.1. Receptor-Mediated Targeting

Ligand–receptor binding becomes a delivery mechanism only when an accessible, disease-biased receptor is coupled to a productive membrane event. Receptor-mediated targeting can support selective binding and, where receptor biology permits, internalization and subsequent trafficking [134]. In cancer, review-level evidence supports strategies involving folate and transferrin receptors, cluster of differentiation 44 (CD44), integrins, epidermal growth factor receptor (EGFR), and human epidermal growth factor receptor 2 (HER2) in receptor-enriched contexts [135]. Direct preclinical vascular evidence comes from vascular cell adhesion molecule 1 (VCAM-1)-directed nanocarriers, which accumulated in inflamed cerebral vasculature and enabled local expression of messenger RNA (mRNA)-encoded protein in mice [155]. In infection, receptor biology can instead support entry blockade, as demonstrated by ACE2-rich membrane nanoparticles against SARS-CoV-2 [151]. These cases represent different mechanisms—uptake or local vascular delivery versus surface-confined entry blockade—and should not be treated as equivalent.

Receptor abundance alone does not predict targeting performance. Accessibility can be limited by glycocalyx shielding, membrane domain segregation, endogenous ligand competition, or rapid recycling, whereas a moderately expressed receptor may remain useful when efficiently internalized or organized within uptake-favorable domains. Receptor-mediated endocytosis links molecular binding to intracellular delivery [134], but surface association, internalization, and productive delivery are not equivalent and should be distinguished experimentally [42]. Performance also depends on ligand presentation, particle properties, receptor organization, and trafficking [42,134]. Receptor targeting is most suitable when accessibility, internalization or transcytosis, and subsequent trafficking align with the objective; it becomes less reliable with heterogeneous or healthy tissue expression or when strong local binding is not accompanied by adequate tissue penetration [122].

#### 5.2.2. Ion Channel and Bioelectric Modulation

Ion channels and membrane potential define functional states that can be modulated but are not nanocarrier targeting determinants by default. Their dysregulation contributes to established channelopathies [137]. In cancer, review-level evidence links channel remodeling to proliferation, migration, and invasion [152], while systematic evidence indicates that pharmacological modulation can alter cancer-relevant phenotypes [153]. These findings support disease relevance and pharmacological actionability, not disease-selective carrier recognition or delivery.

Functional modulation is therefore the principal intervention mode. Channel-directed drugs can alter conductance or downstream electrophysiological signaling, although broad tissue distribution and diverse physiological roles create selectivity and safety constraints [41]. An artificial chloride channel directly disrupted intracellular chloride homeostasis and induced apoptosis in cancer cells through autophagy perturbation [75]. Nanotechnology-based approaches to cancer bioelectricity remain emerging, with a substantial gap between bioelectric mechanisms and translational implementation [156]. Such strategies are most defensible when the abnormal state is disease-enriched or spatially restricted, or when local modulation uses an independently validated targeting mechanism. Channel or membrane potential modulation should otherwise be classified as pharmacological or bioelectric intervention rather than selective carrier targeting.

#### 5.2.3. Lipid-Domain-Associated Interaction and Uptake

Lipid-domain-associated uptake reflects plasma membrane organization rather than recognition of an independent target. Cholesterol-rich rafts and other domains organize receptors and signaling proteins [15], while raft- or caveolae-associated pathways can influence uptake-route selection [154]. In HeLa cells, Chakraborty and Jana showed that attenuating nanoparticle surface charge and adding lipophilic functionality to quantum dot nanoprobes shifted uptake from predominantly clathrin-mediated to lipid raft-mediated endocytosis [157]. The surrounding lipid nanoenvironment can also regulate receptor, channel, and transporter structure and function [40]. Lipid domains are therefore organizational platforms that shape receptor engagement, membrane association, internalization, and signaling rather than stand-alone targeting determinants.

Design relevance requires linkage to a defined interaction or uptake mechanism. Rafts can participate in viral entry in selected infections [158], while lipid-based carrier composition and biophysical properties can be engineered to influence membrane interaction, uptake, and barrier transport [63]. Because domains are dynamic and difficult to define consistently across cell types, diseases, and assays, lipid-domain-associated mechanisms remain context-dependent modulators of engagement and uptake rather than universal selective targeting routes.

#### 5.2.4. Glycocalyx- and Glycan-Mediated Recognition

Glycosylation regulates membrane protein surface presentation and function [52], while cell-surface glycans participate in self, altered-self, and non-self recognition through glycan-binding interactions [53]. Unlike single-receptor recognition, these interactions are often multivalent, spatially distributed, and context-dependent. They contribute to immune-cell recognition [53] and inflammatory adhesion [91], while sialylated glycans can participate in viral attachment [96].

Glycan-associated features are most useful when a defined motif is disease-biased, accessible, and functionally linked to adhesion, immune recognition, or uptake. E-selectin–glycan interactions provide an established example at activated endothelial interfaces [91]. Engineered EVs displaying sialyl Lewis X showed enhanced E-selectin-dependent uptake by activated endothelial cells [159], providing direct evidence that a defined glycan motif can enhance context-dependent recognition and uptake. Glycan-mediated recognition therefore remains conditional on motif accessibility, spatial organization, disease enrichment, and linkage to the intended interaction.

#### 5.2.5. Mechanically Mediated Membrane Interactions and Carrier Physicochemical Properties

Physical membrane interactions arise from coupling between target-membrane state and engineered carrier properties rather than from a single targeting determinant. Theoretical work shows that pre-existing membrane curvature can alter nanoparticle wrapping [160], while membrane curvature, tension, and bending mechanics participate in feedback regulation of trafficking [66]. Carrier surface charge influences membrane association and uptake in a biological-medium-dependent manner [50], and acquired protein coronas can modify nanocarrier identity and interactions [161]. Modeling predicts elasticity-dependent differences in membrane wrapping [49], while experiments show that nanoparticle elasticity can alter the composition and density of cell membrane coatings and thereby modify cellular uptake [162]. Carrier stiffness can also influence elimination half-life, tumor accumulation, and tumor penetration in vivo [23].

These physicochemical variables are context-dependent design parameters, not independent targeting mechanisms. Size, shape, and charge can affect uptake in two-dimensional (2D) cultures differently from accumulation and penetration in three-dimensional (3D) tumor spheroids [163], demonstrating that greater cellular uptake does not necessarily predict deeper tissue penetration. Nanomaterial interactions can also engage innate immune pathways [164]. Physical interactions should therefore be optimized by jointly considering membrane state, carrier mechanics and surface properties, biological-medium effects, tissue penetration, and safety rather than maximizing adhesion or uptake alone.

### 5.3. Biomimetic Membrane-Coated Nanocarriers as Functional Modules

Carrier-borne membrane functions are distinct from disease-associated remodeling of target-cell membranes. The former are source-derived properties retained in or engineered into a carrier shell, whereas the latter describe the pathological interface encountered by the therapeutic system. Synthetic or semisynthetic cores provide structural support, cargo loading, and controlled-release functions, while membrane shells may contribute reduced clearance, vascular adhesion, inflammatory homing, mediator sequestration, homotypic recognition, or antigen display. Membrane source should therefore be selected according to the intended function, disease context, and delivery constraints. The following sections consider RBC, platelet, immune-cell, cancer cell, and hybrid membranes as distinct functional modules.

#### 5.3.1. Red Blood Cell Membrane-Coated Nanocarriers

RBC membrane-coated nanocarriers are prototypical long-circulating biomimetic platforms. A foundational study showed that erythrocyte membranes could be transferred to polymeric nanoparticles and substantially prolong circulation in mice [165]. In native RBCs, cluster of differentiation 47 (CD47)–signal regulatory protein alpha (SIRPα) signaling contributes to self-recognition and limits macrophage-mediated clearance [166]. RBC coatings are consequently developed primarily as circulation- and clearance-modulating interfaces rather than disease-homing membranes. They can also provide decoy functions: RBC membrane-coated nanosponges sequestered staphylococcal α-toxin and reduced toxicity and mortality in mice [167].

Lesion targeting generally requires an additional module. For example, an RBC membrane-coated AD nanotheranostic was functionalized with a transferrin receptor aptamer to provide receptor-directed targeting [168]. RBC coatings alone offer limited lesion selectivity; their principal roles remain circulation support, clearance modulation, and selected decoy functions.

#### 5.3.2. Platelet Membrane-Coated Nanocarriers

Platelet membranes provide adhesion-associated functions linked to vascular injury, thrombosis, inflamed endothelium, and platelet–tumor interactions [169]. In atherosclerosis, platelet membrane-coated rapamycin nanoparticles enhanced plaque homing and reduced progression in apolipoprotein E-deficient (*ApoE−/−*) mice [170], while platelet-coated carriers delivering anti-CD47 antibodies promoted efferocytosis and plaque stabilization [171]. In thrombosis, platelet membrane-coated recombinant staphylokinase improved thrombus-directed delivery and thrombolysis [172]. These examples represent distinct applications of the platelet interface rather than a universal homing mechanism.

Platelet-derived functions are context-dependent and require control of activation state, procoagulant potential, and unintended platelet-associated interactions [169]. As a contextual example of the underlying biology, functionally attenuated platelet decoys inhibited thrombosis and metastatic tumor formation in preclinical models [173]; this finding supports the therapeutic relevance of platelet functions but does not itself validate a membrane-coated carrier.

#### 5.3.3. Macrophage and Neutrophil Membrane-Coated Nanocarriers

Macrophage and neutrophil membrane-coated nanocarriers exploit source-cell functions involved in inflammatory recruitment, adhesion, and mediator binding [149]. In inflammatory arthritis, neutrophil membrane-coated nanoparticles localized to inflamed joints, neutralized proinflammatory mediators, and attenuated synovial inflammation and joint damage [150]. Drug-free neutrophil membrane-coated nanoparticles also showed membrane-dependent anti-inflammatory activity, demonstrating that the shell itself can serve as a decoy or scavenging interface [174].

Macrophage membranes provide complementary functions in macrophage-rich niches. In atherosclerosis, macrophage-biomimetic nanoparticles combined lesion-directed pharmacotherapy with proinflammatory cytokine sequestration [175], while *Mycobacterium*-stimulated macrophage membrane-coated nanoparticles enabled lesion–pathogen dual targeting in preclinical tuberculosis models [176]. Homing, mediator sequestration, and pathogen interaction should nevertheless be demonstrated independently. Source-cell state is a major boundary because macrophage polarization, neutrophil activation, membrane protein retention, and mediator-binding profiles can alter performance [149]. Source phenotype and retained function must therefore be matched to disease context, with immune safety and manufacturing consistency evaluated separately.

#### 5.3.4. Cancer Cell Membrane-Coated Nanocarriers

Cancer cell membranes may transfer tumor-derived cues that support homotypic recognition and phenotype-matched interactions. Surface proteins, adhesion-related molecules, glycoconjugates, and other recognition cues may contribute to preferential interaction with tumor cells sharing related membrane phenotypes [177], as demonstrated in preclinical cancer cell membrane-camouflaged nanoagents [178]. Pan et al. note that homotypic recognition is constrained by intratumoral heterogeneity, variable surface marker expression, and incomplete correspondence between source and target phenotypes [35]. It is therefore most defensible when source and target phenotypes are matched and functionally characterized; preferential tumor-cell interaction does not by itself establish whole-tumor delivery.

Cancer-derived membranes can also provide immunological functions. Cancer cell membrane-coated nanoparticles have combined photodynamic therapy with Toll-like receptor 7 activation to enhance tumor immunotherapy [179], while cancer cell membrane nanodiscs have served as antigen-bearing vaccination platforms [180]. These functions are mechanistically distinct from homotypic recognition: antigen display, antigen-presenting cell engagement, and immune activation do not demonstrate tumor-cell targeting. Antigen presentation claims instead require evidence of antigen retention, antigen-presenting cell engagement, and tumor-relevant immune activation.

Translationally, membrane orientation, coating fidelity, source–target phenotype correspondence, immunogenicity, and manufacturing variability must be controlled [35,148]. Membrane source alone does not establish functional performance; the phenotype supporting the claimed function must remain preserved, accessible, and reproducible after fabrication.

#### 5.3.5. Hybrid Membrane-Coated Systems

Hybrid coatings combine functions that a single membrane source may not provide. Their rationale is deliberate modular pairing rather than presumed multifunctionality: one source may support circulation or reduced clearance, while another provides adhesion, homotypic recognition, or another disease-relevant interaction [181].

An early RBC–platelet hybrid retained markers and functional characteristics from both sources and prolonged circulation, demonstrating the feasibility of combining distinct membrane functions [182]. RBC–cancer cell hybrids subsequently combined RBC-associated circulation support with cancer-cell-derived homotypic recognition in melanoma [183]. More recently, coassembled platelet and erythrocyte membranes combined platelet-mediated recognition of cardiac myofibroblasts and collagen with erythrocyte-associated circulation support for JQ1 delivery in preclinical cardiac fibrosis [184]. These studies support mechanism-matched hybridization, not the assumption that adding membrane sources necessarily improves targeting or efficacy.

The principal boundary is functional compatibility after hybridization. Source ratio, component compatibility, and surface presentation can dilute, mask, or disrupt intended functions and alter off-target recognition or biodistribution. Hybrid coatings are most defensible when each source contributes a defined and functionally attributable role and less justified when functions conflict or the claimed advantage cannot be linked to a specific component.

### 5.4. Engineered EVs, EV Mimetics, and Synthetic–Biological Hybrid Vesicle Platforms

#### 5.4.1. Extracellular Vesicles as Cell-Released Membrane-Bound Delivery Systems

EVs are cell-released, membrane-bound vesicles; operationally defined small EV preparations should not be termed exosomes unless their endosomal origin is demonstrated [185]. Unlike membrane-coated nanocarriers, which transfer isolated membranes onto artificial cores, EVs contain source-derived membrane components and endogenous cargo within the same released vesicular structure. Production conditions can therefore alter both the membrane interface and payload [186]. EVs carry proteins, lipids, and nucleic acids, and their surface composition can influence recipient-cell interactions and uptake [187]. Vesicle-associated transfer of mRNA and microRNA has also been demonstrated experimentally [188].

Therapeutic studies illustrate this delivery potential, although earlier terminology should be interpreted under current EV guidelines. Catalase-loaded vesicles described as exosomes were detected in the brain after intranasal administration and produced neuroprotective effects in a Parkinson’s disease model [189]. This supports the preclinical feasibility of vesicle-associated catalase delivery, not intrinsic tissue tropism or predictable targeting. EV composition, biodistribution, and performance depend on cell source, physiological state, culture and isolation, engineering, cargo loading, and administration route [186]. EVs are therefore biologically instructive vehicles whose membrane identity and cargo function require context-specific qualification.

#### 5.4.2. Engineered EVs for Programmable Targeting and Cargo Delivery

Engineered EVs use surface and cargo modification to make vesicle-mediated delivery programmable. Producer-cell engineering generated brain-directed vesicles displaying a rabies virus glycoprotein (RVG) peptide fused to lysosome-associated membrane glycoprotein 2b (Lamp2b) [190]. Post-isolation click chemistry provides an alternative surface-functionalization route [191], while fragment crystallizable (Fc)-binding EVs demonstrate that modular antibody display can enhance tumor-cell recognition and uptake [192]. Such targeting requires accessible motifs and preserved vesicle integrity, while therapeutic delivery additionally requires productive uptake and downstream cargo activity.

Cargo engineering addresses controlled loading and intracellular release. Exosomes for protein loading via optically reversible protein–protein interactions (EXPLORs) load soluble proteins during vesicle biogenesis and support intracellular delivery [193]. Scaffold engineering can enhance endogenous protein loading, with tetraspanin 2 (TSPAN2) and tetraspanin 3 (TSPAN3) identified as efficient EV-sorting scaffolds [194]. Multimodal engineering has further combined active loading and release with fusogenic endosomal escape to deliver proteins and genome-editing cargoes, including Cas9–single-guide RNA ribonucleoprotein complexes [195]. Evaluation should therefore integrate surface display, vesicle integrity, cargo stability, intracellular release, biodistribution, and therapeutic context rather than rely on targeting or loading efficiency alone.

#### 5.4.3. EV Mimetics and Cell-Derived Nanovesicles

EV mimetics and cell-derived nanovesicles partly address the low yield and manufacturing complexity of naturally secreted EVs [196]. Serial extrusion can generate bioinspired EV-mimetic nanovesicles [197], while microfluidic processing offers a more scalable route to exosome-mimetic nanoparticles [198]. These platforms range from mechanically generated nanovesicles retaining selected source-cell components to engineered mimetics that recapitulate selected EV features with greater formulation and production control [196,197,198]. Their value lies in improving manufacturability while retaining defined functions, not reproducing native EVs exactly.

These products are not biologically equivalent to naturally secreted EVs. Proteomic and post-translational modification profiling identified substantial differences between mechanically generated nanovesicles and exosomes [199]. Direct comparison in a mesenchymal stem cell (MSC)-derived system also revealed differences in molecular cargo and regenerative activity, with extruded nanovesicle profiles more closely resembling parental cells than the corresponding EV preparations [200]. Higher yield therefore does not establish functional equivalence; yield, composition, potency, and safety require independent assessment. Umbilical cord MSC-derived extruded nanovesicles showed no tumorigenic potential under the tested conditions, illustrating the source- and process-specific safety evaluation required for translation [201].

#### 5.4.4. Synthetic–Biological Hybrid Membrane Platforms

Synthetic–biological hybrids combine selected EV functions with the engineering control of synthetic systems. EVs contribute source-dependent membrane interactions with recipient cells, whereas synthetic lipid systems provide greater control over composition, loading, and release. EV–liposome and related hybrids integrate EV-derived components with tunable formulations [202], while EV-inspired synthetic vesicles translate selected biological principles into lipid- or polymer-based carriers rather than reproducing native EVs [3].

Direct studies demonstrate this modular strategy. PEG-mediated fusion of EVs with functionalized liposomes incorporated liposomal components into hybrid vesicular delivery systems [203]. Hybrid vesicles described as exosome–liposome nanoparticles delivered CRISPR/Cas9 systems in MSCs [204], while EV–LNP hybrids enabled functional mRNA delivery [205]. Hybridization is valuable when biological and synthetic modules provide complementary, defined functions and the relevant EV-derived activity remains intact after processing.

#### 5.4.5. Design Boundaries of Engineered EVs, EV Mimetics, and Hybrid Vesicle Platforms

Engineered EVs, mimetics, and hybrids couple biological membrane functions with controllable surface engineering, cargo loading, and release. Membrane origin alone does not confer target specificity or low risk; value depends on alignment among membrane identity, recipient-cell interaction, cargo activity, administration route, and disease context. Major uncertainties include source-cell dependence, heterogeneity, variable loading, route-dependent biodistribution, immune interactions, and manufacturing reproducibility. In hybrids, synthetic components, fusion conditions, and surface display can further alter the biological functions intended to be retained.

These platforms should therefore be evaluated as context-dependent strategies rather than universally targetable carriers. Qualification must distinguish vesicle identity from targeting and therapeutic function and assess membrane display, cargo activity, biodistribution, and safety against the intended mechanism. EV identity and reporting should follow the Minimal Information for Studies of Extracellular Vesicles 2023 (MISEV2023) framework [185], while quality control, safety, and nonclinical evaluation require product-oriented evidence [29,206]. Table 3 links each exploited membrane feature or function to its intervention mechanism, representative evidence, and principal boundary.

**Table 3:** Membrane-guided nanomedicine strategies: representative evidence and design boundaries.

Strategy or Platform	Feature Basis	Representative Contexts	Representative Evidence	Main Boundaries
Receptor-mediated ligand targeting	Disease-biased receptors or antigens; binding, uptake, transcytosis, or decoy action.	Cancer; inflammation; CNS delivery; infection.	Cancer receptor targeting [135]; VCAM-1 vascular targeting [155]; BBB transcytosis mechanisms [207]; ACE2 decoys [208].	Requires accessible targets; binding does not establish productive transport.
Ion channel or bioelectric modulation	Ion channels, membrane potential, and ionic homeostasis; pharmacological or bioelectric modulation.	Cancer; channelopathies; cystic fibrosis.	Channelopathies [137]; chloride channel-induced apoptosis [75]; cancer ion channel and bioelectric pharmacology [152,153].	Broad expression limits selectivity; off-target electrophysiological risk.
Lipid-domain-associated uptake	Raft-associated receptor organization and uptake; viral entry.	Cancer; infection.	Cancer raft signaling [107]; vector uptake [154]; viral entry [158].	Dynamic and context-dependent; not an independent selective target.
Glycocalyx- and glycan-mediated recognition	Defined glycan motifs; immune recognition, inflammatory adhesion, and glycan engineering.	Cancer; inflammation; vascular interfaces.	Tumor glyco-immunology [81]; immune shielding [78]; E-selectin–glycan adhesion [91]; tumor-selective glycan engineering [209].	Motif accessibility, heterogeneity, and normal-tissue overlap limit selectivity.
Physical membrane interactions	Membrane mechanics and carrier charge, elasticity, and geometry; association, wrapping, and penetration.	Cancer; solid-tumor delivery.	Carrier stiffness [23]; nanoparticle–membrane modeling [17]; 3D penetration and charge effects [163].	Stronger interactions may increase nonspecific binding or perturbation without improving penetration.
RBC membrane-coated nanocarriers	RBC membrane proteins and toxin-binding interfaces; clearance modulation and toxin decoy.	Systemic delivery; toxin neutralization.	RBC camouflage [165]; native CD47 self-recognition [166]; toxin nanosponges [167].	Prolonged circulation does not establish lesion homing.
Platelet membrane-coated nanocarriers	Platelet adhesion cues; plaque, thrombus, and circulating tumor-cell interactions.	Atherosclerosis; thrombosis; circulating tumor cells.	Plaque homing [170]; thrombolysis [172]; CTC targeting [210].	Platelet state, procoagulant activity, and tumor-related interactions require control.
Macrophage and neutrophil membrane-coated nanocarriers	Adhesion and mediator-binding proteins; inflammatory homing and sequestration.	Inflammation; atherosclerosis; sepsis; tuberculosis.	Neutrophil decoy [150]; plaque targeting [175]; sepsis sequestration [211]; tuberculosis dual targeting [176].	Source-cell state, retained function, and immune safety require control.
Cancer cell membrane-coated nanocarriers	Tumor-derived recognition cues and antigens; homotypic recognition, antigen display, and immune activation.	Cancer therapy; immunotherapy; vaccination.	Homotypic interaction [178]; combination immunotherapy [179]; vaccination [180]; limitations [35].	Tumor heterogeneity, source–target mismatch, antigen fidelity, and tumor-derived material safety.
Hybrid membrane-coated systems	Complementary source membrane functions; circulation support, adhesion, and homotypic recognition.	Cancer; cardiac fibrosis and heart failure; selected cardiovascular applications.	RBC–platelet hybrids [182]; RBC–cancer cell hybrids [183]; platelet–erythrocyte delivery [184].	Requires controlled source ratios, functional preservation, and compatibility.
Cell-released EV delivery	EV membrane identity and endogenous cargo; recipient-cell interaction and cargo transfer.	Alzheimer’s disease; rheumatoid arthritis.	Quercetin-loaded EVs in AD [212]; miR-22 EVs in AD [213]; M2 macrophage EVs in RA [214].	Source, isolation, cargo, and route constrain performance.
Engineered EV delivery	Surface-targeting motifs and cargo modules; targeted uptake, loading, and intracellular release.	CNS delivery; cancer; protein and RNA delivery; genome editing.	RVG-guided brain siRNA delivery [190]; antibody-guided cancer EVs [192]; EXPLORs protein delivery [193]; genome-editing delivery [195].	Engineering may alter identity and biodistribution; display or loading alone does not establish functional delivery.
EV mimetics and cell-derived nanovesicles	Parental cell membrane features; scalable generation and cargo delivery.	Cancer; regenerative medicine; tissue repair.	Serial extrusion [197]; microfluidic production [198]; nanovesicle–EV comparison [200].	Mechanical generation can alter composition; equivalence to natural EVs cannot be assumed.
EV–liposome and EV–LNP hybrid systems	EV and synthetic lipid components; hybridization, cargo loading, and intracellular delivery.	Nucleic acid delivery; genome editing.	EV–liposome fusion [203]; CRISPR/Cas9 delivery in MSCs [204]; functional mRNA delivery [205].	Hybridization does not ensure retained EV function.

**Note:** The evidence listed is representative rather than exhaustive. Ion channel or bioelectric modulation is not classified as selective nanocarrier targeting without direct evidence of disease-selective delivery. **Abbreviations:** 3D, three-dimensional; ACE2, angiotensin-converting enzyme 2; AD, Alzheimer’s disease; BBB, blood–brain barrier; Cas9, CRISPR-associated protein 9; CD47, cluster of differentiation 47; CNS, central nervous system; CRISPR, clustered regularly interspaced short palindromic repeats; CTC, circulating tumor cell; EV, extracellular vesicle; EXPLORs, exosomes for protein loading via optically reversible protein–protein interactions; LNP, lipid nanoparticle; M2, M2-polarized macrophage phenotype; miR-22, microRNA-22; mRNA, messenger RNA; RA, rheumatoid arthritis; RBC, red blood cell; RNA, ribonucleic acid; RVG, rabies virus glycoprotein; VCAM-1, vascular cell adhesion molecule 1; siRNA, small interfering RNA; MSC, mesenchymal stem cell.

### 5.5. Disease-Context Matching and Therapeutic Scenarios

This section matches membrane-guided designs to major diseases according to membrane feature, access route, and therapeutic objective. Rather than cataloging applications by platform, it evaluates mechanistic fit within each disease context.

#### 5.5.1. Cancer

Cancer illustrates how molecular recognition can become uncoupled from effective delivery. Tumors may present dysregulated receptors [80], glyco-immune remodeling [81], and mucin-rich glycocalyx barriers [78], while acidity, reactive oxygen species, and elevated enzymatic activity provide contextual release triggers [215]. Receptor-mediated strategies are most appropriate when disease-enriched folate or transferrin receptors, CD44, EGFR, HER2, or integrins are accessible and coupled to productive binding and internalization [135]. Receptor engagement nevertheless does not overcome vascular, stromal, and penetration barriers or heterogeneous target accessibility [4,122].

Cancer cell membrane-coated nanoparticles are most relevant when source and target tumors share phenotypes supporting homotypic recognition [35,177]. Pan et al. emphasize that membrane identity alone does not establish selective targeting because intratumoral heterogeneity and variable surface marker expression weaken source–target correspondence [35]. Preclinical studies show that cancer cell membrane camouflage can enhance homotypic interaction and tumor accumulation [178], while programmable coatings can add defined receptor recognition functions to combination therapy [216]. Preferential tumor-cell recognition must be distinguished from whole-tumor delivery, which also requires vascular access, stromal transport, target accessibility, and 3D penetration.

Glycocalyx remodeling has a complementary dual role: tumor-associated glycans may provide recognition cues, whereas a dense mucin-rich glycocalyx can suppress immune-cell engagement [78,81]. Strategies are therefore most defensible when an accessible cue addresses the dominant delivery problem. Membrane heterogeneity, source–target mismatch, poorly characterized glycosylation, and limited 3D penetration reduce predictability [163].

#### 5.5.2. Neurodegeneration

Neurodegenerative diseases pose a distinct delivery problem because access is constrained by BBB transport, central nervous system (CNS) cell-type specificity, and neuroinflammation. In AD, cholesterol-dependent lipid raft organization is associated with Aβ biogenesis [85], while BBB dysfunction and neuroinflammation impose barrier- and tissue-level constraints [86,87]. The BBB is a multicellular barrier rather than an intrinsic membrane property; membrane guidance therefore depends on defined endothelial transport mechanisms within its broader architecture [87,88]. Transferrin and insulin receptors can support transcytosis, but productive brain entry requires endothelial internalization, appropriate trafficking, and abluminal release rather than binding alone [207].

Preclinical studies illustrate complementary strategies. BBB-crossing LNPs enabled systemic mRNA delivery to neurons and astrocytes in mice [217]; RVG-displaying EVs delivered small interfering RNA to multiple brain cell types after intravenous administration [190]; and mannose-coated LNPs enabled microglia-directed microRNA-17 (miR-17) inhibition in an AD mouse model [218]. These examples support mechanism-matched delivery, not intrinsic brain tropism. Translation requires evidence of brain-side release, parenchymal distribution, disease-relevant cell engagement, functional cargo activity, controlled exposure, and neurological safety.

#### 5.5.3. Inflammation

Inflammation creates membrane-guided interfaces through endothelial activation and leukocyte-recruitment cues, while altered permeability modifies access at the barrier level. Unlike tumor-cell recognition, inflammation is a dynamic tissue state rather than a fixed identity. Activated endothelium can upregulate VCAM-1, intercellular adhesion molecule 1 (ICAM-1), and selectins and alter vascular permeability [92]. Increased permeability may facilitate access but does not establish molecular selectivity. VCAM-1-targeted nanocarriers provide direct preclinical evidence of delivery to inflamed cerebral vasculature [155], with utility depending on spatially restricted activation and coupling between adhesion and carrier retention or uptake.

Immune-cell-mimetic membranes provide complementary inflammatory-recognition and mediator-binding functions [149]. In arthritis, neutrophil membrane-coated nanoparticles localized to inflamed joints, sequestered inflammatory mediators, and attenuated synovial inflammation and joint damage [150]. Drug-free counterparts further showed that the membrane shell itself can sequester cytokines and chemokines [174]. These strategies are most defensible when the inflammatory interface is spatially and temporally defined.

#### 5.5.4. Infection

Infectious-disease interfaces depend on pathogen tropism and infection stage. In tuberculosis, coatings derived from *Mycobacterium*-stimulated macrophages enabled dual targeting of tuberculous granulomas and *Mycobacterium tuberculosis*, together with antibacterial photothermal therapy [176]. This exploits functions associated with the infected niche rather than generic membrane targeting.

Viral entry interfaces can instead serve as receptor decoys. ACE2-rich membrane nanoparticles inhibited SARS-CoV-2 infection [151], while human lung-spheroid-cell-derived ACE2 nanodecoys neutralized the virus and reduced lung injury in a nonhuman primate model [208]. Effectiveness remains dependent on receptor use, protease activation, and membrane fusion requirements [95], while some viruses also use sialylated glycans for attachment [96]. Anti-infective design must therefore match the host or pathogen interface to the entry or delivery mechanism while accounting for pathogen variation, inaccessible reservoirs, and host-membrane safety.

#### 5.5.5. Vascular Disease

Vascular nanomedicine operates at blood-exposed interfaces and is jointly influenced by endothelial state, blood constituents, particle properties, and hemodynamics. These factors govern margination, near-wall transport, adhesion stability, endothelial uptake, and in vivo fate [100]. Static binding assays are therefore insufficient to establish vascular targeting under physiological flow. Endothelial activation and plaque inflammation provide membrane-associated interfaces, while platelet adhesion and thrombus formation provide lesion-associated cues. Pathological high shear is a flow-level rather than intrinsic membrane cue, but shear-activated systems can convert it into localized delivery in obstructed vessels [98].

In atherosclerosis and thrombosis, platelet membrane-coated carriers have demonstrated plaque homing and stabilization [170] and thrombus-directed thrombolytic delivery [172], while macrophage-biomimetic nanoparticles combined plaque targeting with proinflammatory cytokine sequestration [175]. These strategies require accessible vascular or immune interfaces that remain functional under relevant flow conditions.

### 5.6. Context-of-Use Framework and Design Boundaries

Membrane-guided nanomedicine should be selected by context of use rather than platform identity. Candidate membrane features or carrier-borne functions—including receptors, glycans, lipid domains, membrane-associated mechanics, and cell-derived recognition interfaces—must match the administration route, carrier architecture, tissue access, and therapeutic objective. A strategy is justified only when its claimed membrane-dependent function addresses a defined disease or delivery barrier.

Specificity and accessibility form the first design boundary. Disease-associated receptors, glycans, and adhesion interfaces may also occur in healthy tissues or non-target lesions, while productive delivery requires physical accessibility and coupling to an appropriate uptake or transport pathway. Biomolecular coronas can mask ligands and alter targeting specificity [25]; receptor binding does not establish productive internalization or trafficking [134]; and tissue-level barriers can limit exposure to the intended interface [4]. Membrane recognition must therefore be evaluated together with downstream delivery.

Immune and blood-contact safety form the second boundary. Biomimicry does not eliminate these risks: EV-based products require product-specific quality and safety assessment [206]; blood-contacting systems require complement and hemocompatibility evaluation [99], and platelet-derived platforms require additional control of platelet-associated effects [169]. Context of use should therefore define the intended interface, dominant barrier, administration route, safety constraints, and measurable membrane-dependent function. Section 6 translates these boundaries into experimental and translational qualification requirements.

## 6. Translational Characterization and Qualification of Membrane-Guided Nanomedicine

**Figure 3:**
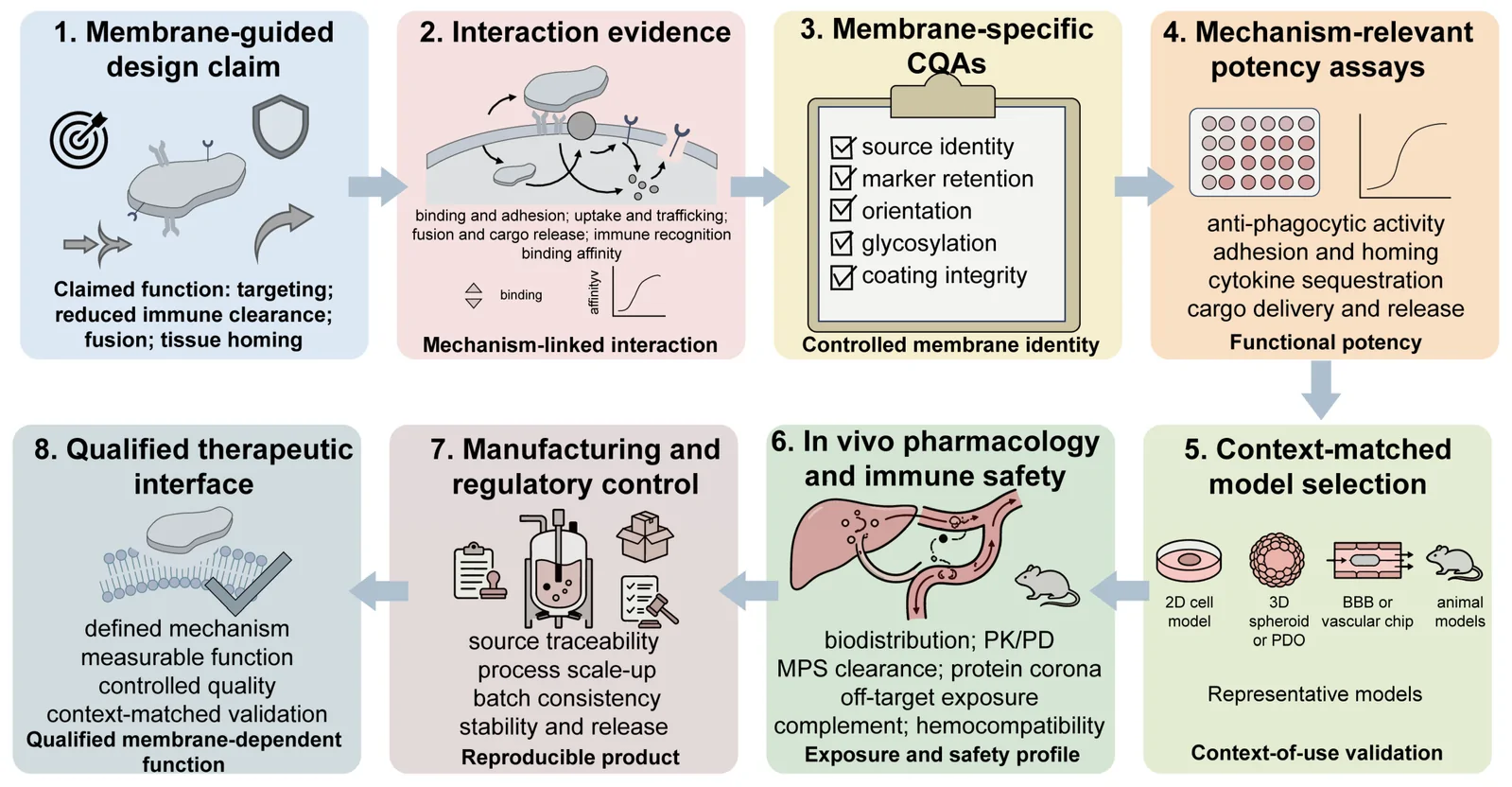
Translational qualification workflow for membrane-guided nanomedicine. This schematic traces a claimed membrane-dependent function—such as targeting, reduced immune clearance, membrane fusion, or tissue homing—from a membrane-guided design claim through linked qualification domains comprising mechanism-linked interaction evidence, membrane-specific critical quality attributes, mechanism-relevant potency assays, context-matched model selection, in vivo pharmacology and immune safety, and manufacturing and regulatory control. A qualified therapeutic interface requires concordance among a defined mechanism, measurable function, controlled product quality, context-of-use validation, and reproducible performance rather than descriptive membrane mimicry alone. 2D, two-dimensional; 3D, three-dimensional; BBB, blood–brain barrier; CQAs, critical quality attributes; MPS, mononuclear phagocyte system; PDO, patient-derived organoid; PK/PD, pharmacokinetics and pharmacodynamics.

The preceding sections frame cell membrane biophysics as a therapeutic interface linking disease-associated remodeling to membrane-guided interventions and disease-context matching. However, membrane-guided design alone does not confer therapeutic reliability. Translational qualification requires the claimed membrane-dependent function to be experimentally demonstrated, quantitatively measured, controlled, and linked to pharmacological performance. This requires a connected framework spanning interaction evidence, function-related product quality, potency, context-matched validation, in vivo performance, and manufacturing and regulatory control. Figure 3 summarizes the progression from design claims to qualified therapeutic interfaces.

### 6.1. Characterizing Membrane–Therapeutic Interactions

Characterizing membrane–therapeutic interactions determines whether a membrane-guided strategy performs as intended. The central question is not whether a nanocarrier is detected near or inside cells, but whether its interaction is specific, productive, compatible with the intended safety profile, and mechanistically linked to the claimed function. Complementary methods interrogate different interface properties: FRAP assesses membrane mobility, and AFM assesses structure and mechanics [219,220]; nuclear magnetic resonance reports membrane dynamics, and EIS reports interfacial electrical behavior [221,222]; and flow cytometry quantifies cell- and marker-associated signals [223].

When the proposed mechanism involves adsorption, insertion, or structural perturbation, spatially resolved methods should determine both particle location relative to the bilayer and perturbation depth. QCM-D monitors real-time adsorption-associated frequency and dissipation changes at supported interfaces; neutron reflectometry provides depth-resolved information on particle localization, bilayer structure, and hydration; and AFM force spectroscopy assesses nanoscale morphology and mechanical stability. Their combined application to supported lipid bilayers showed that predominantly surface-associated nanoparticles can produce charge-dependent perturbations extending into the hydrophobic region and toward the inner leaflet, together with increased hydration, defect formation, and reduced nanomechanical stability [62]. These methods distinguish particle position from the depth and consequences of membrane perturbation and should complement cell-based uptake and functional delivery assays. Method selection should follow the claimed function—defined binding, productive uptake, fusion or cargo release, altered permeability or mechanics, or immune recognition—rather than provide isolated endpoints.

Binding and adhesion must be established at the cell membrane. Nanomaterials can associate through electrostatic, hydrophobic, or ligand–receptor-mediated contacts [5,6], while protein coronas can reshape the exposed biointerface, mask ligands or membrane motifs, and alter specificity [25,161]. Increased target-cell fluorescence should not be interpreted as mechanism-specific targeting unless nonspecific adsorption, serum-protein effects, and receptor-independent binding are excluded. For receptor- or glycan-mediated targeting, ligand blocking, receptor knockdown or knockout, competition assays, and orthogonal affinity measurements can distinguish molecular recognition from nonspecific adsorption.

Equilibrium affinity does not define the temporal behavior of membrane engagement. When kinetics are mechanism-relevant, association and dissociation rates, receptor residence time or membrane association lifetime, and time-resolved adhesion should be quantified. Surface plasmon resonance can resolve binding kinetics; in multivalent systems, valency can prolong receptor residence [46], whereas strong multivalent adhesion can generate receptor-limited, long-lived out-of-equilibrium states [47]. Kinetic persistence must therefore be interpreted against the intended membrane-level function rather than assumed to improve targeting or delivery. This is particularly important for membrane-derived or membrane-coated systems, in which homotypic adhesion, inflammatory tropism, or immune-cell interactions inferred from membrane source must be verified as functional binding events [148].

The next interaction to resolve is internalization, intracellular trafficking, and functional cargo delivery. Cellular uptake is not equivalent to therapeutic delivery: a carrier may remain surface-associated, enter through a nonproductive route, accumulate in endosomes or lysosomes, or release cargo too late for pharmacological activity. Endocytic pathway assignment should combine genetic or pharmacological perturbation, live-cell imaging, endosomal or lysosomal colocalization, ultrastructural analysis, and quantitative trafficking readouts rather than rely on pharmacological inhibitors alone [42,43]. For intracellular RNA, protein, or genome-editing cargoes, endosomal escape and functional release must be distinguished from uptake; engineered EV studies demonstrate the value of cargo-activity readouts for confirming productive delivery [195].

Membrane contact may also produce fusion, controlled permeability modulation, biophysical remodeling, or injurious disruption. Lipid composition, sterol content, and phase behavior influence membrane interactions and permeability [57], but vesicle proximity does not establish fusion. Förster resonance energy transfer-based approaches can report lipid mixing [223] or nanomedicine integrity and fate [224], but orthogonal functional readouts are required to confirm productive fusion. Permeability, leakage, and barrier integrity assays, including EIS where relevant, can help distinguish therapeutic transport from membrane or barrier disruption [51], while hemolysis and cytotoxicity assays distinguish therapeutic modulation from injury or formulation-related toxicity [20]. Immune-interface characterization should assess relevant innate responses [164] and, for blood-contacting systems, complement activation, platelet and coagulation interactions, and hemocompatibility [99,224]. These interaction-level readouts support membrane-specific critical quality attributes (CQAs) and mechanism-relevant potency assays. When membrane remodeling is itself the claimed mechanism, time-resolved measurements should establish the rate, persistence, and reversibility of changes in membrane organization, mechanics, permeability, or fusion rather than rely solely on endpoints.

### 6.2. Critical Quality Attributes and Potency Assays

Following characterization of membrane–therapeutic interactions, CQAs must be defined to preserve the claimed membrane-dependent function. CQAs should extend beyond general nanoparticle descriptors and be prioritized according to functions such as prolonged circulation, vascular adhesion, inflammatory homing, homotypic recognition, cargo delivery, membrane fusion, or immune modulation.

General physicochemical attributes remain essential baseline controls. Particle size, polydispersity index, zeta potential, morphology, colloidal stability, drug loading and release, and serum and storage stability support formulation consistency and batch comparability. For lipid-based vesicles, phase behavior, membrane permeability, and fluidity also influence leakage, fusion propensity, and release kinetics. However, these measurements do not establish membrane-guided function: stable size and surface charge may coexist with loss of membrane protein orientation, glycan recognition, coating integrity, fluidity, or biological activity. Conventional physicochemical characterization is therefore a prerequisite for qualification, not proof of a qualified membrane interface.

Membrane-specific CQAs should be selected from function-linked attributes such as source identity and cell state, membrane protein and lipid composition, glycosylation, membrane orientation, coating completeness and integrity, membrane-to-core ratio, contaminants, and stability [148]. For cell membrane-coated nanoparticles, fabrication, characterization, targeting design, and translational constraints should be considered together [148,225]. Nanoparticle elasticity can affect coating formation and subsequent nano–bio interactions [162], while coating degree can alter internalization [226].

EV-based products require additional CQAs because vesicle identity, cargo composition, and biological activity are coupled. Relevant attributes include vesicle source, purity, heterogeneity, surface markers, lipid and glycan profiles, cargo identity and stability, and contaminants. Target-cell interaction and functional cargo activity should be assessed separately as mechanism-relevant readouts. MISEV2023 provides a reporting framework for EV source, production, separation, characterization, and nomenclature [185], but does not by itself establish functional delivery or mechanism-relevant potency. Product quality and potency must therefore be linked to the expected mechanism of action [29,206].

Mechanism-relevant potency assays bridge membrane-specific CQAs and therapeutic activity. They should distinguish biologically active products, detect process-related changes, and reflect the intended mechanism [29,206]. Each assay should directly test the claimed function, such as anti-phagocytic activity, vascular adhesion, mediator sequestration, homotypic recognition, antigen retention, cargo delivery, membrane fusion, or controlled release. When one readout is insufficient, complementary assays should capture distinct function-linked outcomes. Platform-specific examples are summarized in Table 4. Section 6.3 evaluates whether these readouts remain informative in context-matched preclinical models.

### 6.3. Preclinical Models and Context-of-Use Validation

Preclinical validation of membrane-guided nanomedicine should be organized by context of use rather than model complexity alone. After membrane interactions, CQAs, and potency assays are defined, the question is whether these readouts remain informative in models representing the relevant biological barrier and therapeutic setting. 2D cultures, 3D spheroids, patient-derived organoids (PDOs), immune co-cultures, organ-on-chip systems, and animal models address different translational questions rather than forming a simple hierarchy. Dynamic barrier or flow-dependent questions may require appropriately engineered organ-on-chip models.

For tumor-targeted strategies, model selection should distinguish cellular recognition from tissue-level delivery. The 2D tumor-cell assays can test receptor expression, blocking, homotypic adhesion, and initial uptake but cannot establish solid-tumor delivery. Spheroids assess penetration and 3D distribution [163], whereas PDOs add patient-specific architecture and molecular heterogeneity. For strategies claiming altered immune-cell engagement or reduced phagocytic clearance, tumor–immune co-cultures or immune-containing organoids can capture interactions absent from tumor-only models [227]. Whole-organism models remain necessary for assessing biodistribution, systemic toxicity, and pharmacological efficacy and should be interpreted alongside human-relevant models.

For BBB and neurodelivery strategies, validation should distinguish endothelial binding, transcytosis, abluminal release, parenchymal distribution, and cell-specific delivery. Endothelial monolayers and transendothelial electrical resistance measurements provide initial information on barrier integrity and formulation effects but lack the BBB’s full cellular architecture and hemodynamic environment. BBB-on-chip models better represent barrier organization and enable spatial analysis of nanoparticle transport across vascular and perivascular compartments [228]. Productive receptor-mediated transcytosis must be demonstrated rather than inferred from endothelial binding [207]. Microglia-containing brain organoids and related multicellular systems provide a context for assessing CNS cell-type interactions and microglial responses [229]. Brain entry alone does not establish cell-specific cargo activity or long-term neurological safety.

Inflammatory and immune-targeting strategies require models of dynamic disease states rather than static target expression. Cytokine-stimulated endothelial models can test VCAM-1- or ICAM-1-dependent adhesion [92], as illustrated by VCAM-1-targeted nanocarriers [155]. However, acute stimulation does not fully reproduce chronic inflammatory or fibrotic states in which ECM remodeling and tissue mechanics reshape immune-cell localization and function [123,124]. Models should reproduce the inflammatory state underlying the claimed membrane-dependent interaction.

Infection models should reproduce the relevant host–cell context, pathogen tropism, or intracellular niche. Differentiated human airway epithelium provides a physiologically relevant model of epithelial infection and viral tropism [93], while genetically engineered organoids can identify host factors required for coronavirus infection [94]. When intracellular phagocyte reservoirs are central to the therapeutic claim, macrophage-based models are more appropriate, as illustrated by tuberculosis-directed membrane-coated nanoparticle studies [176].

Vascular and thrombotic strategies require blood flow, shear, and relevant blood-contact conditions. Static endothelial or platelet assays can screen initial adhesion but cannot establish vascular targeting under physiological or pathological hemodynamics. Flow chambers and vascular organ-on-chip systems assess margination, endothelial adhesion, detachment resistance, and delivery under controlled shear [100,230]. Claims of shear-triggered release or thrombus-directed delivery require models reproducing the relevant pathological flow and blood components rather than extrapolation from static binding [98]. Platelet activation, coagulation, and hemocompatibility should be assessed when mechanistically relevant.

Context-of-use validation links interaction evidence and CQA–potency readouts to disease-relevant models without replacing subsequent evaluation of biodistribution, pharmacokinetics, immune clearance, and organ-level toxicity. Table 4 presents the platform-specific qualification matrix.

**Table 4:** Function-centered qualification matrix for membrane-guided nanomedicine platforms.

Platform Class	Claimed Membrane Function	Function-Linked CQAs	Potency Assays	Context-of-Use Models	Qualification Boundaries
Receptor- or glycan-directed nanocarriers [155,207,209]	Receptor or glycan recognition; target-cell binding; uptake or transcytosis.	Ligand identity, density, orientation, accessibility, and retention.	Blocking or competition; receptor perturbation; target-positive versus target-negative binding and uptake; transcytosis or cargo activity.	Target-positive and target-negative 2D cells; 3D spheroids or PDOs; barrier models.	Accumulation alone does not establish specificity or productive transport.
RBC membrane-coated nanoparticles [165,166]	Reduced phagocytic clearance; prolonged circulation.	RBC source identity; CD47 retention and accessibility; membrane-to-core ratio.	Macrophage phagocytosis; CD47 blocking, where mechanistically claimed.	Macrophage and blood-contact models.	Coating does not establish anti-phagocytic activity; prolonged circulation does not prove lesion targeting or blood-contact safety.
Platelet membrane-coated nanoparticles [99,100,169,170,172]	Vascular adhesion; plaque or thrombus binding; lesion-directed delivery.	Platelet source identity and activation state; adhesion marker retention and accessibility.	Adhesion under flow; plaque or thrombus binding; cargo activity or thrombolysis.	Flow-based vascular models; plaque or thrombus models.	Static adhesion does not establish flow-dependent targeting; coagulation and hemocompatibility require separate assessment.
Macrophage and neutrophil membrane-coated nanoparticles [150,174,175,176,211]	Inflammatory homing; mediator sequestration; infection-site targeting.	Source-cell identity and activation state; adhesion- and mediator-binding protein retention.	Mediator sequestration; adhesion or homing; lesion or pathogen targeting; anti-inflammatory or anti-infective activity.	Inflamed-endothelium and immune cell models; inflammatory or intracellular infection models.	Source-cell state may affect retained function; uptake alone does not establish homing or potency.
Cancer cell membrane-coated nanoparticles [35,177,178,216]	Homotypic recognition; preferential tumor-cell interaction; tumor-directed delivery.	Tumor-cell source identity; recognition marker retention, orientation, and accessibility.	Homotypic binding; matched versus mismatched tumor-cell binding and uptake; cargo activity.	Matched and mismatched tumor-cell models; 3D spheroids or PDOs; orthotopic or xenograft models.	Phenotype mismatch, intratumoral heterogeneity, and limited penetration constrain homotypic recognition and whole-tumor delivery.
Natural EVs [185,186,188,214]	Source-dependent recipient-cell interaction; cargo transfer and modulation.	EV source identity; purity and heterogeneity; marker, lipid, glycan, and cargo profiles; contaminants.	Recipient-cell interaction and uptake; cargo delivery and activity; source-relevant response.	Source- and recipient-cell-matched models; disease-relevant barrier and immune models.	Markers or particle counts do not establish functional cargo delivery.
Engineered EVs [190,191,192,193,194,195]	Programmable surface display; target-directed delivery; functional intracellular cargo delivery.	Display motif density and accessibility; vesicle identity and integrity; cargo loading and stability; engineered component retention.	Target-cell binding and uptake; cargo delivery and activity.	Target-cell-matched models; disease-relevant barrier or tissue models.	Engineering may alter vesicle identity and biodistribution; display or loading alone does not establish functional delivery.
Cell-derived nanovesicles [196,197,198,199,200,201]	Parental membrane feature retention; recipient-cell interaction; cargo delivery.	Source identity; membrane protein retention; vesicle integrity and purity; cargo and contaminant profiles; compositional consistency.	Recipient-cell interaction and uptake; cargo delivery; retained source-cell bioactivity.	Parental cell and natural EV comparators; recipient-cell-matched disease models.	Mechanical generation may alter composition and function; equivalence to natural EVs cannot be assumed.
EV–liposome and EV–LNP hybrid vesicles [202,203,204,205]	Retention of EV-derived surface features; enhanced cargo loading; functional cargo delivery.	Hybridization efficiency; EV-to-synthetic-lipid ratio; EV marker retention and accessibility; vesicle integrity; cargo loading and stability; unfused components.	Retained target-cell interaction; cargo delivery and activity.	Target-cell-matched models; disease-relevant 3D or barrier models.	Membrane mixing does not establish stable hybrid formation or preserved EV identity and function.

**Note:** Coating integrity and stability are shared CQAs for membrane-coated systems. In vivo PK, biodistribution, and immune or blood-contact safety should be assessed according to the claimed function and context of use. **Abbreviations:** 2D, two-dimensional; 3D, three-dimensional; CD47, cluster of differentiation 47; CQAs, critical quality attributes; EV, extracellular vesicle; LNP, lipid nanoparticle; PDOs, patient-derived organoids; PK, pharmacokinetics; RBC, red blood cell.

### 6.4. In Vivo Barriers and Pharmacological Behavior

In vivo barriers mark the transition from preclinical validation to pharmacological performance. In vitro binding, uptake, fusion, or potency does not ensure predictable in vivo behavior [231]. After administration, membrane-guided platforms encounter blood proteins, immune cells, endothelial barriers, ECM, tissue flow, clearance systems, and intracellular trafficking barriers. These are contextual determinants rather than intrinsic membrane properties. Evaluation should determine how membrane identity, biological environment, tissue transport, release kinetics, and dose–response relationships jointly shape in vivo performance.

Biodistribution and off-target accumulation are primary barriers. Membrane camouflage, ligand modification, or EV-based design may prolong circulation or enhance lesion interaction but cannot confer absolute specificity. Protein adsorption and uptake by the mononuclear phagocyte system (MPS) can redirect carriers to clearance organs and alter systemic exposure [232,233]. Prolonged circulation must be demonstrated rather than inferred from membrane source because carrier properties and host biology jointly determine biodistribution and clearance [231]. Effective delivery therefore requires quantitative whole-body biodistribution, organ exposure, and off-target toxicity data.

Immune and blood-contact interactions require separate qualification. Protein coronas can mask membrane-associated recognition cues or ligands, alter their accessibility, and redirect cellular uptake [25,161]. Biomimetic origin does not eliminate innate immune recognition or cytokine responses [164]. Complement activation and hemocompatibility require direct evaluation [99]; hemolysis, platelet activation, and coagulation should also be assessed when relevant to the platform and administration route. Repeated dosing can alter clearance, as illustrated by anti-PEG-associated accelerated blood clearance of PEG-containing LNPs [234]. Risks should be evaluated for the intended route, dosing schedule, and patient-relevant context.

Biological barriers determine whether membrane recognition produces therapeutic exposure. Carriers must reach the appropriate tissue compartment, cross the dominant barrier, and release active cargo. BBB binding should be distinguished from transcytosis, parenchymal distribution, and cell-specific uptake [207,217], while tumor-cell targeting remains limited by transvascular transport, ECM diffusion, and interstitial pressure [122,163]. Infection can add mucus, biofilm, or intracellular niche barriers [176,235]; inflammation may involve spatially diffuse activation [92,149]; and vascular targeting remains flow-dependent [98,100]. Recognition must therefore produce sufficient tissue exposure, persistence, cargo release, and intracellular activity.

Pharmacokinetics and pharmacodynamics (PK/PD) integrate these barriers into therapeutic performance. Circulation half-life, organ distribution, lesion retention, carrier degradation, cargo release, intracellular trafficking, and immune clearance determine exposure–response relationships. Bilayer properties can influence permeability, leakage, and release kinetics in lipid-based vesicles [63]. For EVs, labeling artifacts can confound biodistribution measurements [236], while heterogeneity and product-specific clearance complicate pharmacokinetic interpretation [29,206]. For membrane-coated nanoparticles, coating integrity can alter internalization behavior [226]. Administered dose and tissue accumulation must therefore be distinguished from bioavailable cargo, intracellular exposure, and therapeutic effect.

### 6.5. Function-Centered Manufacturing Control and Reproducibility

Manufacturing and product control are integral to qualification when biological activity depends on a membrane-derived interface [237]. The central question is whether source selection, membrane preparation, purification, coating or fusion, cargo loading, and storage preserve the feature responsible for the intended function rather than merely producing a nominally biomimetic particle. EV process development and quality control should remain linked to expected biological activity [29,206]. For membrane-coated nanoparticles, function-linked control should address coating formation and integrity, including elasticity-dependent coating behavior [162], the effects of coating degree on internalization [226], and broader preparation and translational constraints [225]. Manufacturing control must therefore demonstrate functional preservation, not only physicochemical consistency.

Source and process variability are major manufacturing boundaries. Source-cell state and culture conditions can influence membrane or vesicle identity, cargo, and biological activity [186,196]. Production methods, including microfluidic generation, can also affect scalability, consistency, and therapeutic performance [198]. Mechanically generated nanovesicles may differ from naturally secreted EVs in protein composition, post-translational modifications, cargo, and biological activity [199,200]. Purification should control non-vesicular, biological, and process-related impurities while preserving function-linked product attributes [29,206]. For hybrid vesicles, processing should control hybridization efficiency and residual unfused or free components [202,203].

Scale-up and storage should be evaluated for changes in product attributes and function rather than particle appearance alone. For lipid-based systems, bilayer properties influence permeability and leakage [63], whereas EV storage conditions can affect vesicle integrity, surface markers, cargo stability, and biological activity [238]. Release criteria should therefore combine membrane-specific product attributes with function-linked readouts appropriate to the claimed mechanism.

Regulatory evaluation should focus on product definition and reproducible function rather than biomimetic labeling. Product identity, purity, potency, safety, and consistency should be defined and reproducibly controlled in relation to the intended context of use [237]. Favorable preclinical targeting or pharmacology does not ensure a reproducible, clinically developable product. Table 5 compares representative translational trajectories.

### 6.6. From Descriptive Membrane Mimicry to Qualified Therapeutic Interfaces

This framework reframes membrane-guided nanomedicine as a qualification problem rather than descriptive mimicry. Natural membrane origin alone does not confer therapeutic value, and cell membrane-coated, EV-derived, or other biomimetic carriers are not translationally mature unless their membrane-dependent function is demonstrated. That function must be mechanistically defined, quantitatively measured, validated in the intended context of use, preserved during manufacturing, and linked to pharmacological performance. This is consistent with industry perspectives emphasizing reproducible characterization and product control for nanomedicines [229]. The central question is not whether a platform resembles a biological membrane, but whether its claimed function is measurable, controllable, and reproducible.

Qualification should form an evidence chain rather than a collection of independent steps. Mechanistic definition must connect to function-relevant CQAs and potency assays; these readouts must remain informative in context-matched models; and the resulting activity must translate into interpretable in vivo exposure, pharmacological response, and acceptable safety. Manufacturing must preserve the same function with adequate batch-to-batch consistency. Failure at any point separates descriptive biomimicry from a qualified therapeutic interface. Claims must therefore remain bounded by the biological context, administration route, and barrier environment in which the function was demonstrated.

Regulatory and clinical translation require alignment among mechanism, product definition, reproducible quality, and therapeutic performance. Product identity, purity, potency, safety, and consistency should be controlled relative to the intended mechanism and context of use, with product-specific attributes aligned with applicable regulatory requirements [231]. Table 5 shows that attrition can occur at distinct pharmacological, clinical, manufacturing, or strategic stages. A clinically credible membrane-guided strategy must maintain a measurable, reproducible, and pharmacologically meaningful membrane-dependent function throughout qualification.

## 7. Conclusions and Outlook

Cell membrane biophysics provides a framework for understanding why nanomedicines succeed or fail at cellular and tissue interfaces. Beyond a selectively permeable boundary, the membrane is a dynamic therapeutic interface governing ligand recognition, adhesion, endocytosis, fusion, trafficking, immune recognition, and material–cell compatibility. This perspective shifts the field from a formulation-centered to an interface-centered view: performance depends on how engineered materials engage biological membrane interfaces and how disease-associated membrane states shape transport and biological responses. Rather than organizing the field by carrier identity, this framework connects disease-associated membrane states to mechanistically defined opportunities, design boundaries, and experimentally qualifiable functions.

Disease-associated remodeling provides both rationale and constraint. Changes in lipid metabolism, fluidity, mechanics, receptor organization, glycocalyx architecture, and immune recognition motifs can create candidate therapeutic entry points or delivery-relevant cues across cancer, neurodegeneration, inflammation, infection, and vascular disease, whereas altered ion channel activity and bioelectric states more often support pharmacological or functional modulation. These alterations are heterogeneous, dynamic, and mechanistically distinct. Accessible, disease-enriched receptors or glycans may support preferential recognition when coupled to productive uptake or transport. In contrast, lipid domains, ion channel and bioelectric states, and mechanical phenotypes remain context-dependent modulators or design cues unless directly linked to selective delivery. Therapeutic relevance therefore requires accessibility, disease enrichment, functional linkage, and consistency across relevant patients, disease stages, and treatment conditions.

Membrane-guided strategies must therefore be defined by context of use. Membrane-coated nanoparticles, engineered EVs, hybrid platforms, receptor-directed carriers, and lipid-domain-associated strategies can provide meaningful advantages only when their membrane-dependent functions match the intended disease biology. Platforms derived from RBCs, platelets, immune cells, cancer cells, and EVs illustrate distinct functions, including prolonged circulation, vascular adhesion, inflammatory homing, homotypic recognition, antigen display, and cargo transfer. No platform is universally targeted or intrinsically safe simply because it is membrane-derived or biomimetic.

Translational qualification remains the central challenge. Membrane-level mechanisms must be connected to interaction evidence, function-relevant CQAs and potency assays, context-matched models, and in vivo evaluation of biodistribution, corona formation, immune recognition, pharmacokinetics, intracellular release, and long-term safety. Manufacturing must preserve function-linked membrane and cargo attributes with adequate batch consistency while meeting scalability and regulatory requirements.

Future progress will require integrating membrane biophysics with patient-relevant models, multi-omics membrane profiling, quantitative biointerface characterization, organ-on-chip and humanized systems, and computational design. These approaches should distinguish membrane phenotypes that support testable design rules from associative or context-limited observations. A key objective is to determine when measurable equilibrium membrane properties can quantitatively predict transport kinetics, thereby converting descriptive states into predictive, context-specific design rules. The actionable output is not another catalog of membrane-derived platforms, but a pathway from claimed membrane-dependent functions to mechanistic evidence and translational qualification. Through this interface-centered framework, nanomedicine can become more mechanistically grounded, contextually validated, and translationally credible.

## Figures and Tables

**Table 1:** Biophysical determinants of membrane–therapeutic interactions in membrane-guided nanomedicine.

Determinant	Key Membrane Variables	Mechanistically Linked Events	Design Implications	Main Boundaries
Lipid fluidity and domain organization	Composition; leaflet asymmetry; acyl chain saturation; sterol content; phase state; lipid–protein coupling; rafts and nanodomains [14]	Receptor organization and signaling [22]	Match surface chemistry; assess protein adsorption and uptake [24]	Context-dependent; coronas may mask ligand accessibility [25]
Membrane mechanics and deformability	Tension; stiffness; viscoelasticity; deformability; membrane–cortex coupling [16,48]	Modeled deformation and wrapping [49]	Evaluate carrier mechanics in relation to wrapping [49] and in vivo disposition [23]	Mechanical effects are cell- and context-dependent [16,48]
Electrochemical properties and ion channel regulation	Membrane potential; ion gradients; ion channel activity [41]; surface charge [50]	Ion flux and signaling; channel modulation [41]; electrostatic interactions [50]	Measure zeta potential in relevant media [50]; use EIS for mechanism-relevant electrical readouts [51]	Charge effects are context-dependent and do not imply selective targeting [50]
Glycocalyx and immune recognition	Glycocalyx architecture; surface glycans; glycosylation; recognition motifs [10,52]	Self, altered-self, and non-self recognition [53]; innate immune signaling [54]; immune interactions [55]	Preserve motif accessibility; account for protein adsorption and corona formation [24,56]	Recognition is motif- and context-dependent [53]; coronas may redefine biological identity [56]

**Note:** This table provides a mechanism-oriented index; the cited references are representative rather than exhaustive. **Abbreviations:** EIS, electrochemical impedance spectroscopy.

**Table 2:** Disease-associated membrane remodeling, candidate entry points, and delivery barriers.

Disease Context	Dominant Membrane Remodeling	Functional Consequences	Candidate Therapeutic Entry Points	Delivery and Translational Boundaries	Evidence Level and Representative Evidence
Cancer	Receptor dysregulation; lipid-domain remodeling; aberrant glycosylation and glycocalyx thickening; altered membrane-coupled cell mechanics.	Proliferative and invasive signaling; immune evasion; altered receptor organization and therapeutic response.	Accessible receptors; glycan–lectin interfaces; lipid-domain cues; tumor-surface recognition patterns.	Tumor heterogeneity and variable membrane states; glycocalyx shielding; normal-tissue overlap; receptor and glycan inaccessibility.	Human-cell mechanics evidence [70]; review-level lipid, receptor, and glyco-immune evidence [79,80,81]; direct glycocalyx evidence [78].
Neurodegeneration	Lipid dysregulation; cholesterol-dependent domain remodeling; Aβ–membrane interactions.	Synaptic dysfunction; Aβ–membrane injury; neuroinflammation.	Lipid microenvironment and Aβ–membrane interaction modulation; endothelial transport interfaces.	BBB crossing does not ensure parenchymal distribution or neuronal delivery; limited CNS cell engagement; neuroimmune safety.	Human lipid evidence [82]; molecular dynamics Aβ–membrane evidence [83]; broader synthesis [84,85,86]; BBB evidence [87,88]; direct selective delivery validation limited.
Inflammation	Glycocalyx shedding; endothelial activation and E-selectin upregulation; glycan remodeling; immune-cell membrane cues.	Increased permeability; leukocyte rolling, adhesion, and recruitment; altered immune-cell trafficking.	Inflamed-endothelium adhesion; E-selectin–glycan interfaces; leukocyte-recruitment cues.	Stage-, tissue-, and vascular-bed dependence; off-target immune modulation; acute, chronic, and fibrotic contexts.	Mechanistic human-cell glycan–adhesion evidence [89]; endothelial and inflammatory synthesis [90,91,92].
Infection	Host-receptor and protease availability; fusion-relevant membrane context; glycan receptor availability.	Pathogen entry and tropism; epithelial infection and injury; intracellular replication.	Receptor- and protease-dependent entry blockade; pathogen-attachment and glycan-mediated interfaces.	Pathogen and variant dependence; broad receptor expression; coreceptor availability and fusion context; intracellular access; host-membrane safety.	Human airway evidence [93]; gene-edited organoid causal evidence [94]; fusion and entry reviews [32,95]; glycan–receptor synthesis [96].
Vascular disease	Endothelial glycocalyx injury and adhesion remodeling; plaque lipid remodeling; platelet-surface cues; altered mechanosensing.	Plaque inflammation and lipid heterogeneity; thrombosis; altered permeability; flow-dependent adhesion.	Accessible endothelial, plaque, platelet, and thrombus interfaces under defined flow.	Hemocompatibility, complement, and coagulation risks; flow and lesion-stage heterogeneity; adhesion instability or detachment.	Human plaque-lipid association [97]; mouse shear-triggered delivery [98]; multispecies blood-contact safety [99]; vascular, glycocalyx, and flow synthesis [72,90,100].

**Note:** Entries summarize dominant, nonexclusive disease-context relationships. Tissue-, barrier-, and flow-level processes are treated as consequences or delivery constraints rather than intrinsic membrane remodeling. Representative evidence does not imply validation of every candidate entry point for selective nanocarrier delivery. **Abbreviations:** Aβ, amyloid-β; BBB, blood–brain barrier; CNS, central nervous system.

**Table 5:** Representative translational trajectories and attrition points relevant to membrane-guided nanomedicine.

Representative Case	Design Premise	In Vivo Pharmacology and Safety	Manufacturing Controls	Clinical Status and Translational Lesson
Doxil (PEGylated liposomal doxorubicin) [239,240,241,242]	PEGylation prolongs circulation and alters drug disposition; no active targeting.	Low clearance and limited volume of distribution; formulation-specific toxicities.	Liposome composition and size; doxorubicin encapsulation; PEG surface; release profile.	FDA approved; benchmark for nontargeted liposomal delivery.
CALAA-01 [243,244,245,246]	TfR-directed delivery of *RRM2* siRNA.	Rapid clearance; tumor localization and RNAi proof of mechanism; immune reactions and DLTs.	Transferrin–PEG ligand integrity; four-component assembly; siRNA loading; component ratio.	Phase I terminated; tumor delivery and RNAi proof of mechanism did not ensure continued development.
BIND-014 [247,248,249,250]	PSMA-directed docetaxel delivery.	Prolonged nanoparticle-associated docetaxel exposure; preclinical tumor enrichment; manageable clinical toxicity.	PSMA-ligand display; particle size and composition; docetaxel loading and release.	Phase II in mCRPC completed; development was discontinued despite activity as the treatment landscape evolved.
MM-302 [251,252,253,254]	HER2-directed liposomal doxorubicin delivery.	Prolonged circulation; heterogeneous tumor deposition; limited cardiac toxicity in phase I.	Anti-HER2 scFv display; liposome integrity; doxorubicin encapsulation and retention.	Phase II HERMIONE stopped for futility; targeting and early activity did not ensure benefit over comparator therapy.
exoASO-STAT6 (CDK-004) [255,256,257]	PTGFRN-enriched EV delivery of *STAT6* ASO to tumor-associated macrophages.	Preclinical myeloid cell enrichment, *STAT6* knockdown, and antitumor activity.	PTGFRN display; ASO loading; EV identity and integrity.	Phase I terminated during corporate restructuring; clinical efficacy was not established.

Note: Function-linked CQAs and potency assays are summarized in Table 4; Table 5 emphasizes downstream pharmacology, manufacturing, clinical outcomes, and strategic attrition. Clinical status was verified against regulatory, trial registry, or company records available as of August 2026. **Abbreviations:** ASO, antisense oligonucleotide; CQAs, critical quality attributes; DLTs, dose-limiting toxicities; EV, extracellular vesicle; FDA, U.S. Food and Drug Administration; HER2, human epidermal growth factor receptor 2; mCRPC, metastatic castration-resistant prostate cancer; PEG, polyethylene glycol; PSMA, prostate-specific membrane antigen; PTGFRN, prostaglandin F2 receptor inhibitor; RNAi, RNA interference; *RRM2*, ribonucleotide reductase regulatory subunit M2; scFv, single-chain variable fragment; siRNA, small interfering RNA; *STAT6*, signal transducer and activator of transcription 6; TfR, transferrin receptor.

## Data Availability

No new data were created or analyzed in this study. Data sharing is not applicable to this article.

## Abbreviations

The following abbreviations are used in this manuscript:

2D	two-dimensional
3D	three-dimensional
Aβ	amyloid-β
ACE2	angiotensin-converting enzyme 2
AD	Alzheimer’s disease
AFM	atomic force microscopy
BBB	blood–brain barrier
Cas9	CRISPR-associated protein 9
CD44	cluster of differentiation 44
CD47	cluster of differentiation 47
CFTR	cystic fibrosis transmembrane conductance regulator
CNS	central nervous system
CQA	critical quality attribute
CRISPR	clustered regularly interspaced short palindromic repeats
ECM	extracellular matrix
EGFR	epidermal growth factor receptor
EIS	electrochemical impedance spectroscopy
EV	extracellular vesicle
EXPLORs	exosomes for protein loading via optically reversible protein–protein interactions
Fc	fragment crystallizable
FRAP	fluorescence recovery after photobleaching
GPCR	G protein-coupled receptor
HER2	human epidermal growth factor receptor 2
ICAM-1	intercellular adhesion molecule 1
LNP	lipid nanoparticle
MISEV2023	Minimal Information for Studies of Extracellular Vesicles 2023
MPS	mononuclear phagocyte system
mRNA	messenger RNA
MSC	mesenchymal stem cell
PD-1	programmed cell death protein 1
PD-L1	programmed death-ligand 1
PEG	polyethylene glycol
PK/PD	pharmacokinetics and pharmacodynamics
PDO	patient-derived organoid
QCM-D	quartz crystal microbalance with dissipation monitoring
RBC	red blood cell
RNA	ribonucleic acid
RVG	rabies virus glycoprotein
SARS-CoV-2	severe acute respiratory syndrome coronavirus 2
Siglec	sialic acid-binding immunoglobulin-like lectin
SIRPα	signal regulatory protein alpha
TAZ	transcriptional coactivator with PDZ-binding motif
VCAM-1	vascular cell adhesion molecule 1
YAP	Yes-associated protein
